# Fragment-Based Discovery of Fenamic Acids as Pan-Species Inhibitors of the Soluble Epoxide Hydrolase Phosphatase Domain

**DOI:** 10.1021/acschembio.6c00502

**Published:** 2026-07-28

**Authors:** W. Felix Zhu, Guillaume Feugray, Steffen Brunst, Hind Messaoudi, Meng Yang, Noemi Martinez-Conde, Andreas Krämer, Lilia Weizel, Johanna M. H. Ehrler, Jan S. Kramer, Astrid Kaiser, Mohammad Al-Tarrass, Tony Pereira, Manon Valet, Valéry Brunel, Manfred Schubert-Zsilavecz, Stefan Knapp, Felix F. Lillich, Victor Hernandez-Olmos, Thomas Duflot, Kerstin Hiesinger, Hongwu Qian, Anna Proschak, Jeremy Bellien, Ewgenij Proschak

**Affiliations:** † Institute of Pharmaceutical Chemistry, 9173 Goethe University, Frankfurt am Main 60438, Germany; ‡ Inserm U1096 EnVI, Univ Rouen Normandie, Rouen F-76000, France; § Department of Biochemistry, CHU Rouen, Rouen F-76000, France; ∥ Department of Cardiology, The First Affiliated Hospital of USTC, MOE Key Laboratory for Membraneless Organelles and Cellular Dynamics, Hefei National Research Center for Interdisciplinary Sciences at the Microscale, Division of Life Sciences and Medicine, 12652 University of Science and Technology of China, Hefei 230027, China; ⊥ Laboratory of Medicinal Chemistry (CSIC Associated Unit), Faculty of Pharmacy and Food Sciences, and Institute of Biomedicine (IBUB), University of Barcelona (UB), Barcelona E-08028, Spain; # Structural Genomics Consortium (SGC), Buchmann Institute for Molecular Life Sciences (BMLS), Frankfurt am Main 60438, Germany; ¶ Department of Pharmacology, CHU Rouen, Rouen F-76000, France; ∇ CIC-CRB 1404, CHU Rouen, Rouen F-76000, France; ○ 587462 Fraunhofer Institute for Translational Medicine and Pharmacology ITMP, Frankfurt am Main 60596, Germany

## Abstract

Targeting the N-terminal phosphatase domain of soluble epoxide hydrolase (sEH) could potentially represent a pharmacological strategy for treatment of cardiometabolic diseases. However, development of chemical tools to investigate the therapeutic potential of sEH phosphatase (sEH-P) inhibition has proven challenging due to varying activities between different species isoforms and lack of selectivity toward the C-terminal hydrolase domain, which exhibits the epoxide hydrolase activity (sEH-H). Herein, we describe the fragment-based discovery of FZ581 (**49**), an sEH-P inhibitor against human, rat, and mouse isoforms. In vivo studies with this compound in mice and rats demonstrated a significant increase of main sEH-P substrate levels without affecting plasma levels of sEH-H substrates. Altogether, we provide a pan-species sEH-P inhibitor to study the pharmacotherapeutic potential of sEH-P inhibition.

## Graphical abstract

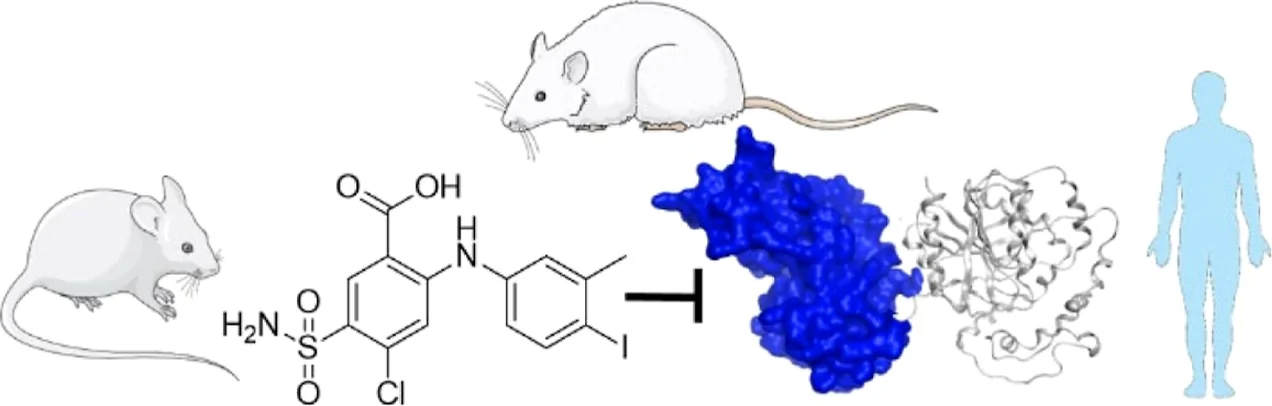

## Introduction

Soluble epoxide hydrolase (sEH) is a bifunctional enzyme that is involved in lipid signaling. The C-terminal domain of sEH (CTD) exhibits epoxide hydrolase activity (sEH-H) and converts epoxidized polyunsaturated fatty acids (PUFAs) and its derivatives toward the corresponding vicinal diols.^1^ It is a well-established pharmacological target with compounds inhibiting sEH-H currently being under clinical investigation for the treatment of pain^2^ and inflammation^3^ as well as subarachnoid hemorrhage^4^ and insulin resistance.^5^ Besides sEH-H, the N-terminal domain of sEH (NTD) possesses lipid phosphatase activity (sEH-P).^6,7^ sEH-P converts several natural and artificial phosphate species,^8^ and recent studies indicate that lysophosphatidic acids (LPAs) are the endogenous substrates of sEH-P. Initially, Oguro and Imaoka showed that sEH-P converts LPAs in vitro,^9^ followed by Morisseau et al., who demonstrated that sEH-P contributes to LPA dephosphorylation in liver and lung tissues.^10^ Allelic variants of sEH possess different activities toward LPAs.^11^ Leuillier et al. reported that rats with CRISPR/Cas9-mediated knock-in of inactive sEH-P exhibited significantly higher levels of LPAs, thereby confirming that sEH-P is involved in their degradation in vivo. Importantly, this was associated with protective effects against the development of obesity as well as against cardiac ischemia-reperfusion injury, supporting the interest of inhibiting sEH-P for the treatment of cardio-metabolic diseases. In the olfactory bulb of mice, which were deficient in sEH-P activity, Oguro et al. observed an increase of 2-arachidonoyl-LPA with a concomitant decrease of 2-arachidonoyl-glycerol (2-AG) levels, confirming that physiologically sEH is involved in LPA conversion.^12^

The validation of sEH-P as a potential drug target relies on the availability of pharmacological tools. One of the first reported lipid-like inhibitors of sEH-P was *N*-acetyl-*S*-farnesyl-l-cysteine **1** (Figure 1).^13,14^ **1** mimics farnesyl pyrophosphate, which has been reported as a sEH-P substrate by Tran et al.^15^ Our group reported the first orally available inhibitor SWE101 (**2**), which is a suitable pharmacological tool to investigate the role of sEH-P in vivo.^16^ However, while **2** is a potent inhibitor of human and rat sEH-P, it lacks activity toward the mouse isoform. This is consistent with other reports, where inhibitory activity can significantly vary between species.^17^ Given the fact that mouse is the most widely used disease model organism, novel pharmacological tools exhibiting selective activity across different species are needed. Recently, Kihara et al. reported that 3-amino-4-hydroxy benzoic acid **3** inhibits human and mouse isoforms at micromolar concentrations.^18^ Liu et al. demonstrated that **3** modulates the turnover of LPA and other phospholipids in mouse ex vivo.^19^ These findings support the hypothesis that it is possible to develop a pan-species sEH-P inhibitor. The aim of this study was the development of a tool compound that inhibits human, mouse, and rat sEH-P, enabling the investigation and validation of sEH-P as a potential pharmacotherapeutic target.

**Figure 1:**
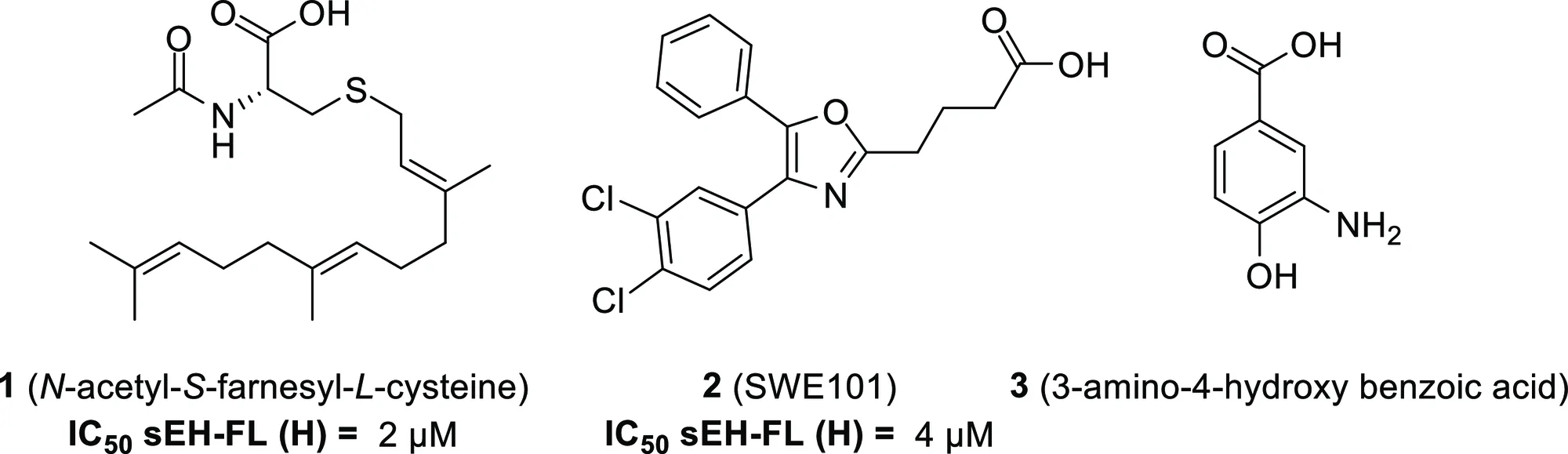
Previously published sEH-P inhibitors.

## Results

### Fragment-Based Hit Generation

To identify a starting point for a pan-species sEH-P inhibitor, we performed a fragment screening using NTDs of both mouse and human sEH. Recombinantly expressed proteins were subjected to differential scanning fluorimetry (DSF) and a 480-membered library of drug-derived fragments was screened against both sEH-P isoforms (Figure 2). Considering a shift in melting temperature (Δ*T*_m_) of >1 °C as a potential binding event, we were able to identify two fenamic acid derivatives **4** and **5** as the most promising hits with *T*_m_s of >2 °C against both isoforms. We subsequently validated the activity of the hits in a fluorescence-based enzymatic assay. We determined an IC_50_ of 39 μM for the methylated compound **4**, while the chloro substituted analogue **5** exhibited an IC_50_ of 15 μM. Calculation of ligand efficiency (LE) confirmed that this scaffold represents a well-suited starting point for the optimization of pan-species sEH-P inhibitors.^20^

**Figure 2:**
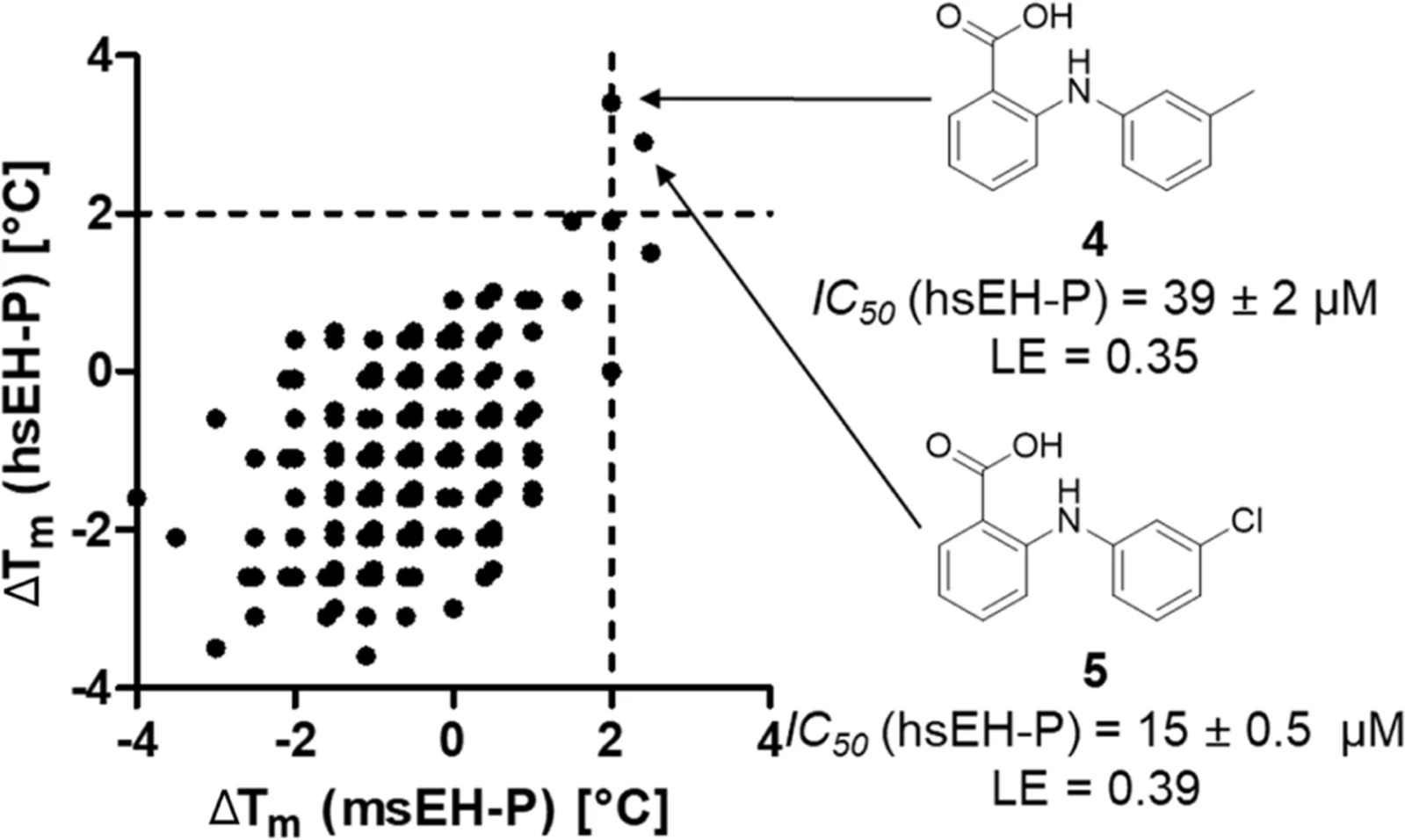
DSF screening of the Prestwick drug-derived fragment library against human and mouse sEH-NTD. Hits with a Δ*T*_m_ ≥ 2 °C are shown on the right with the corresponding IC_50_ for inhibition of human sEH-P (NTD) obtained in the orthogonal enzyme activity assay and corresponding LE values.

### Synthesis

To enable the structure–activity relationship study of fenamic acids against the sEH-P, synthesis of most fenamic acid derivatives was achieved by Ullmann–Goldberg coupling between 2-bromobenzoic acids and amines under microwave irradiation (Scheme 1a). Generally, it was also possible to perform this reaction under microwave-free conditions by using DMF instead of EtOH as the solvent and heating the reaction mixture in an oil bath. As sulfonamide- and iodine-substituted derivatives were not obtainable under either of these conditions, they were synthesized by nucleophilic aromatic substitution of 2-fluorobenzoic acids with the corresponding amines and LiHMDS instead (Scheme 1b). Compound **20** was obtained by the nucleophilic aromatic substitution of 2-chloroquinoline by anthranilic acid (Scheme 1c). All amines were commercially available except for naphthalene derivative **54**, which was synthesized by copper-catalyzed halide exchange from the corresponding bromide (Scheme 1d). Tetrazole **52** was obtained by Buchwald–Hartwig coupling of 2-bromobenzonitrile with 3-chloroaniline, followed by 1,3-dipolar cycloaddition (Scheme 1e,f). Methylation of **5** was achieved using MeI (Scheme 1g).

**Scheme 1:**
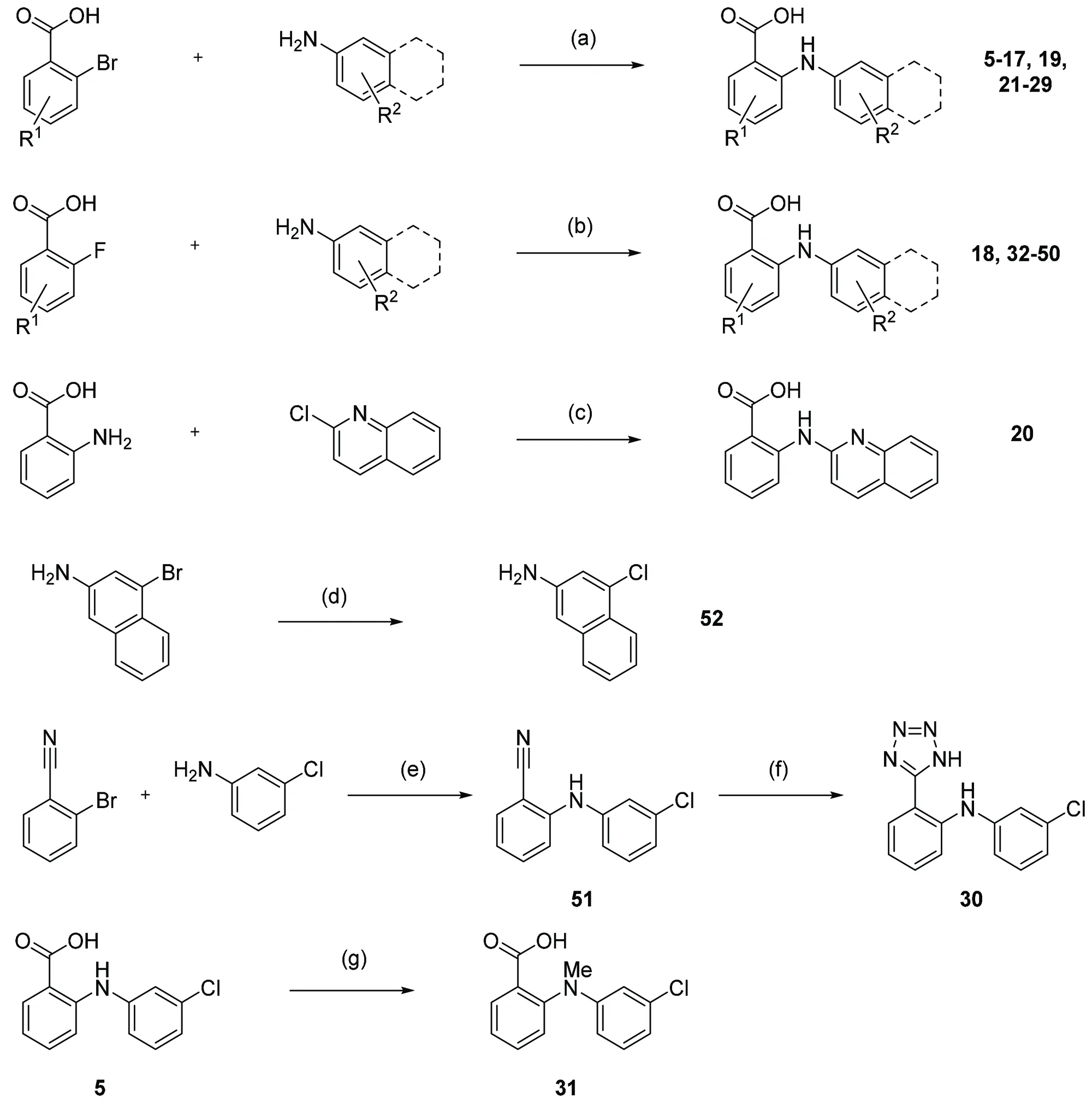
General Synthesis of Fenamic Acid Derivatives^a^

a Reagents and conditions: (a) K_3_PO_4_, Cu­(OAc)_2_, EtOH[Fn s1fn1], 110 °C (MW), 0.5 h; (b) LiHMDS, THF, −78 °C to reflux, 2 h; (c) DIPEA, EtOH, 50 °C, o.n.; (d) TBACl, Cu_2_O, (*S*)-proline, EtOH, 130 °C, 15 d; (e) Pd_2_(dba_3_), *rac*-BINAP, NaOMe, toluene, 90 °C, o.n.; (f) Tributyltin­(IV) azide, *p*-xylene, 150 °C, o.n.; (g) MeI, NaH, DMF, 4 h, rt.

### SAR Studies

The inhibitory activity against sEH-P was assessed by a previously reported, fluorescence-based enzyme activity assay.^21^ In this kinetic assay, the conversion of the fluorogenic fluorescein diphosphate (FDP) by the recombinant NTD is monitored, allowing to determine the IC_50_ of the inhibitors. The chemical modifications mainly focused on the introduction of substituents at the two aryl moieties. Starting from the fragment-based hit **5**, a fenamic acid derivative with an IC_50_ of 14.9 μM, substituents were first introduced on ring A at position 4 and 5 (Table 1). Both electron donating and electron withdrawing substituents were systematically introduced at different positions. Thereby, electron donating substituents like methyl and methoxy were not tolerated (**6–9**). Considering electron withdrawing substituents, both –F and –Cl abolished activity when introduced in the position 5 (**10** and **12**). In position 4, –F was tolerated (**11**) and –Cl improved activity (**13**). A nitrile group was also better tolerated at position 4 than 5 (**14, 15**). On the contrary, introducing a –CF_3_ group at position 5 was more beneficial than at position 4 (**16, 17**). Finally, introduction of a sulfonamide in position 5 in combination with beneficial –Cl in position 4 led to the most potent derivative **18**.

**Table 1:**
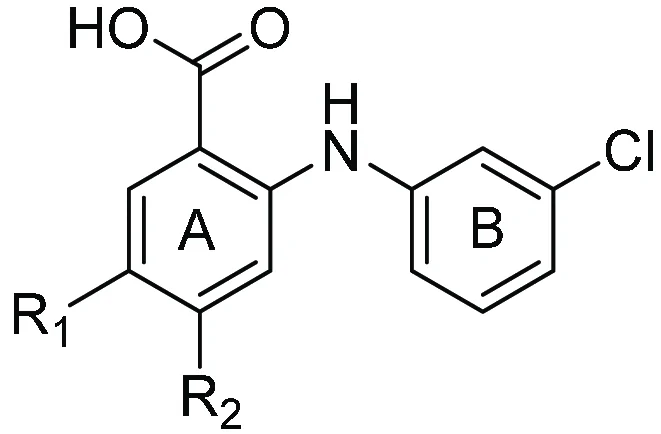
Variation of Substituents on Ring A^a^

cmpd	*R*_1_	*R*_2_	IC_50_ [μM]
**5**	–H	–H	15 ± 0.5
**6**	–CH_3_	–H	inactive
**7**	–H	–CH_3_	inactive
**8**	–OMe	–H	inactive
**9**	–H	–OMe	inactive
**10**	–F	–H	inactive
**11**	–H	–F	22 ± 4
**12**	–Cl	–H	inactive
**13**	–H	–Cl	6.6 ± 0.5
**14**	–CN	–H	22 ± 3
**15**	–H	–CN	13 ± 0.4
**16**	–CF_3_	–H	4 ± 0.4
**17**	–H	–CF_3_	6 ± 0.7
**18**	–SO_2_NH_2_	–Cl	3 ± 0.4

a IC_50_ for inhibition of human sEH-NTD are expressed as the mean ± standard deviation (SD) (*n* = 3, triplicates).

Next, we explored the SAR of ring B (Table 2). Introduction of heterocycles such as pyridine, quinoline, or indole resulted in loss of activity (**19–21**). Moreover, a fluorine scan^22^ in ortho-, meta-, and para-positions of ring B (compounds **22–24**) led to a decrease of activity, revealing a sterical rather than an electronic importance of the meta-chlorine substituent. Shifting –Cl from the meta-to the ortho-position led to a potency decrease (compound **25**), but para–Cl in compound **26** was equally tolerated. While replacement of –Cl by –CH_3_ in the meta-position led to decreased inhibitory activity (compound **4**), the same replacement in the para-position was well tolerated (**27**). Finally, introduction of a 1-naphthyl moiety instead of 3-chlorophenyl led to decreased activity (**28**), while the 2-naphthyl-substituted compound **29** was the most potent from this series.

**Table 2:**
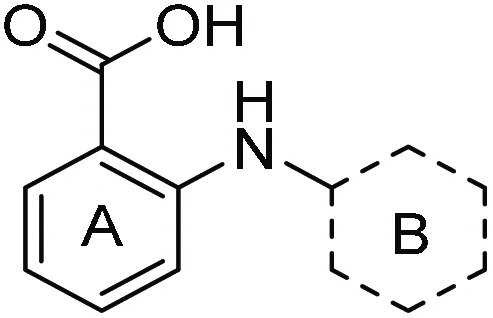

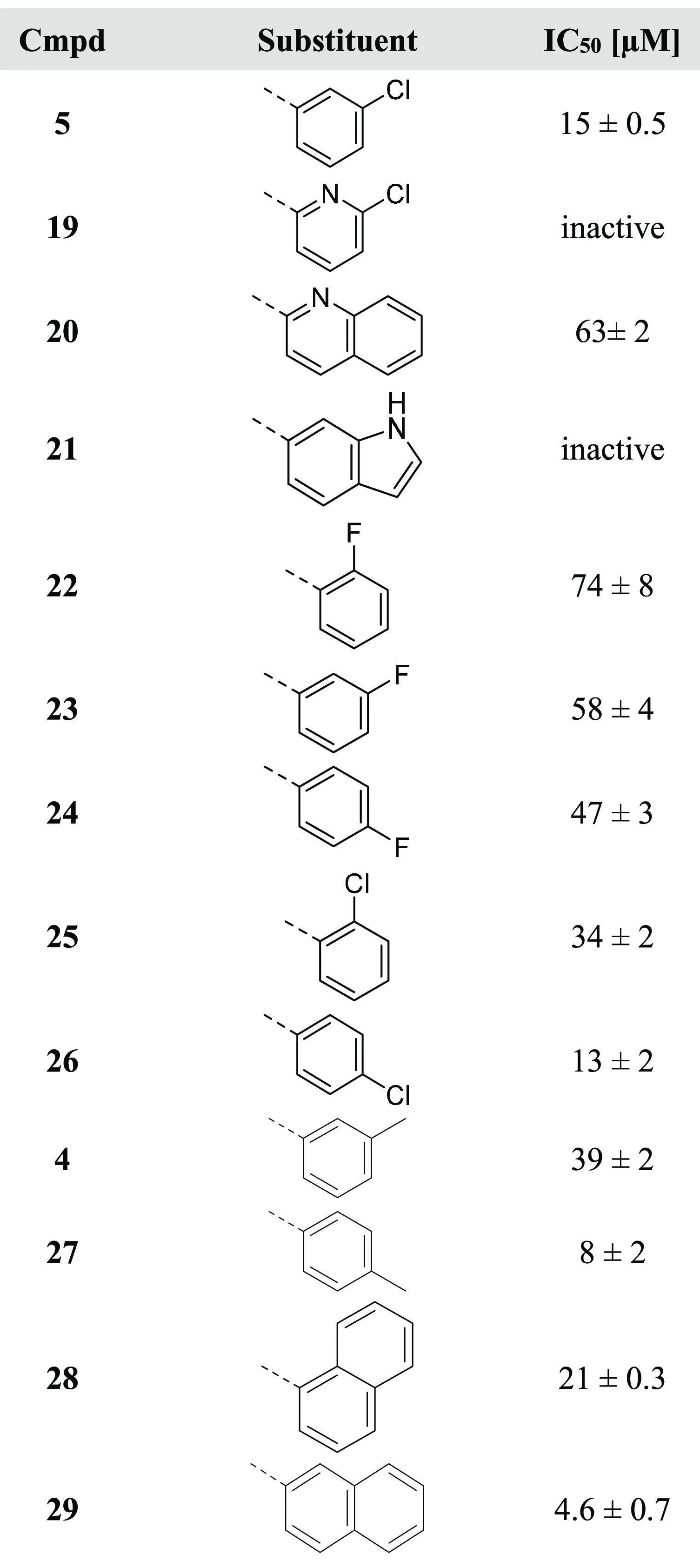
Variation of Ring B^a^

a IC_50_ for inhibition of human sEH-NTD are expressed as the mean ± SD (*n* = 3).

Besides the introduction of substituents at the two aryl rings, the bioisosteric substitution of the carboxylic acid group by a tetrazole (**30**) and the methylation of the amino group (**31**) were evaluated (Table 3). While methylation abolished the inhibitory activity, the introduction of the more lipophilic and less acidic tetrazole slightly improved the activity.

**Table 3:**
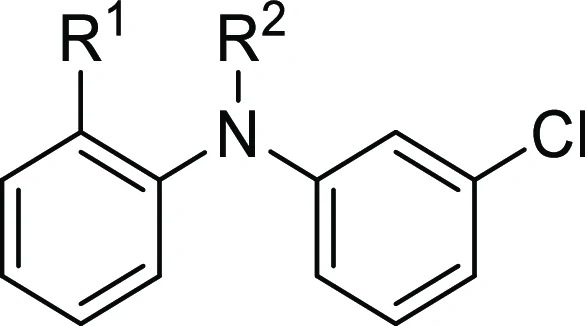

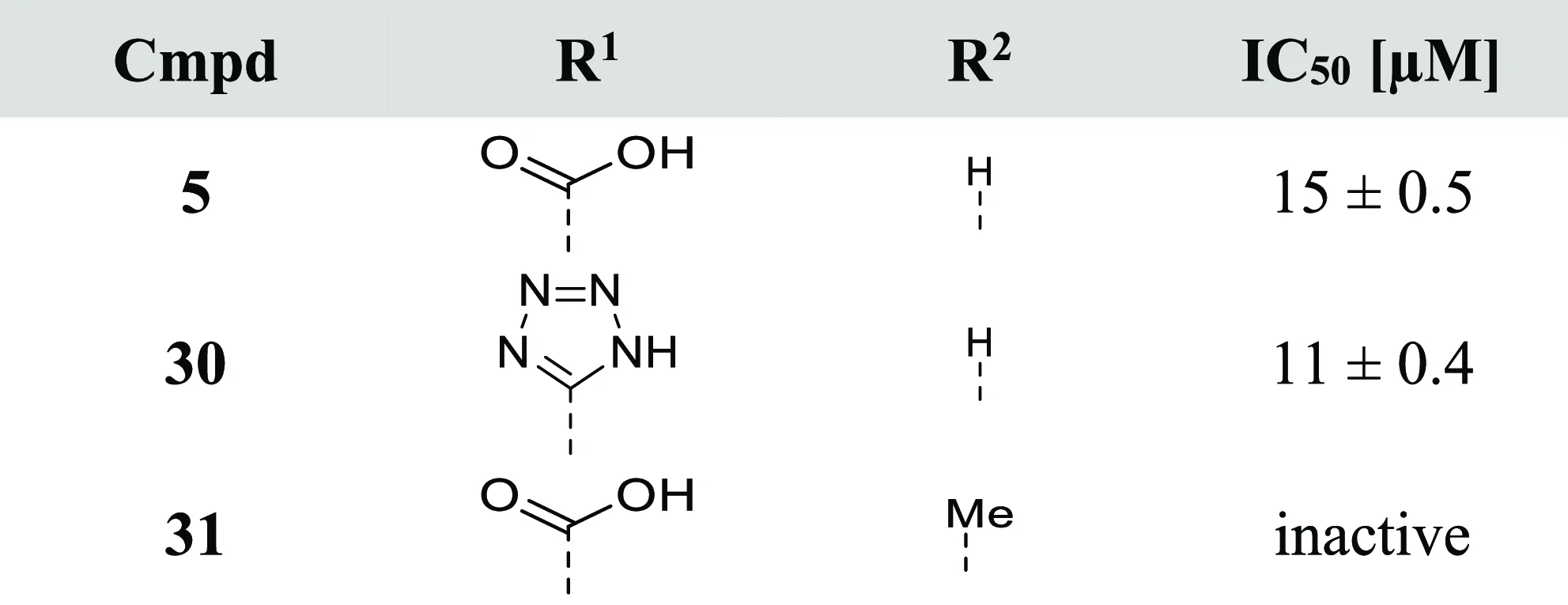
Other Derivatives^a^

a IC_50_ for inhibition of human sEH-NTD are expressed as the mean ± SD (*n* = 3, triplicates).

In the last optimization round (Table 4), we combined the substitution pattern of the most active compound from ring A (compound **15**) with the most promising amine fragments (Table 2). Matsumura et al. recently demonstrated that the substrates of sEH-P affect sEH-H activity and vice versa.^23^ Therefore, the inhibition against sEH-H was also determined, as previous studies have shown that inhibitors of sEH-P often also inhibit sEH-H.^16^ Starting with the most potent 2-naphthyl substituent, the resulting compound **32** exhibited a 9-fold improvement of activity against human sEH-P compared to **29** and it also inhibited the murine sEH-P with an IC_50_ of 1.9 μM. Introduction of chlorine (**33**) and bromine (**34**) in the 4-position of the naphthyl substituent led to further improvement in activity. However, the naphthyl-substituted derivatives were also active against sEH-H in both species.

**Table 4:**
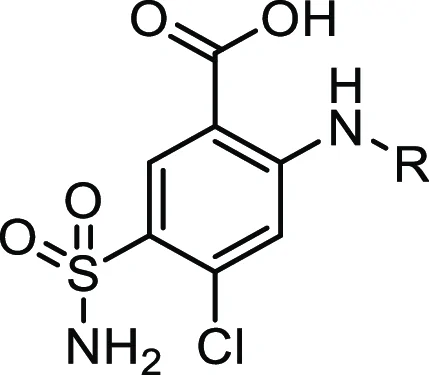

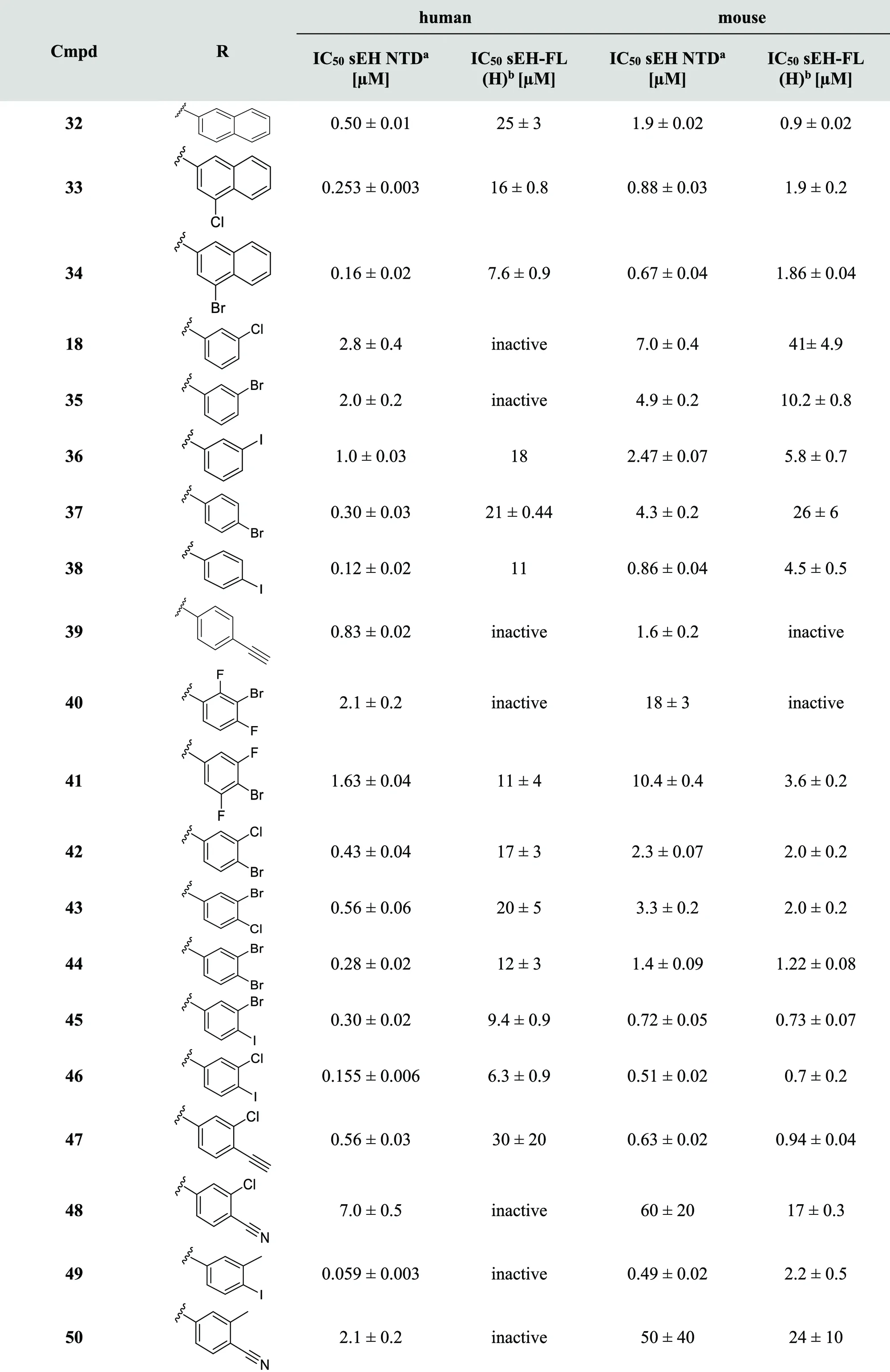
Sulfonamide Series—Comparison Between Human and Mouse sEH

a Phosphatase activity of the isolated N-terminal domains.

b Hydrolase activity of the full-length sEH protein. IC_50_ are expressed as the mean ± SD (*n* = 3, triplicates).

We therefore returned to the initial 3-chlorophenyl series, which was inactive against human sEH-H (compound **18**). Exchange of the 3–Cl substituent by larger halogens like bromine (compound **35**) and iodine (compound **36**) led to a slight potency increase. Interestingly, whereas switching the chlorine from 3- to 4-position had almost no effect on potency before (compound **5** vs compound **26**), a robust potency increase was observed for bromine (compound **35** vs compound **37**) and iodine (compound **36** vs compound **38**). The introduction of an alkyne as an isostere of iodine (compound **39**) was tolerated, while sEH-H inhibition was abolished. Introduction of two fluorines in the ortho-position of the bromine (compounds **40** and **41**), which aimed to further enhance potential σ-hole interactions,^24^ did not improve activity. Several combinations of two halogen substituents in the 3- and 4-position (**42**–**46**) were well tolerated but did not lead to significant improvement of potency. The combination of the sEH-P/sEH-H selective 4-alkyne with the potency-improving 3-chlorine (compound **47**) did not retain its selectivity against the murine sEH. When introducing 4-nitrile, which is sterically similar but electronically different to 4-alkyne (compound **48**), a significant potency loss could be observed. However, the combination of a methyl group next to the 4-iodine substituent (**49**) yielded the most active inhibitor of sEH-P with a 2-fold gain of activity compared to compound **38**. Notably, compound **49** was inactive against human sEH-H. Lastly, like with compound **48**, replacement of 4-iodine by 4-nitrile also led to potency loss (compound **50**).

Compound **49** was selected as the most promising compound of this series due to its potent activity toward the NTD of sEH and selectivity over the sEH-H. Therefore, its full activity profile was determined, including inhibition for human, mouse, and rat sEH-FL (P) (Table 5). In a previous study, we observed differences in the inhibitory activity of sEH-P inhibitors between the isolated NTD and the full-length enzyme.^16^ This was also the case for compound **49**. However, it is to our knowledge the only reported compound that robustly inhibits the sEH-FL (P) across all three species in the single-digit micromolar range. While it is inactive against the human sEH-H, it inhibits the hydrolase activity of mouse and rat sEH.

**Table 5:** Full Characterization of the Inhibitory Activity of Compound **49** against sEH Enzymes from Different Species

	human sEH			mouse sEH			rat sEH	
cmpd	IC_50_ sEH-NTD^a^ [μM]	IC_50_ sEH-FL (P)^b^ [μM]	IC_50_ sEH-FL (H)^c^ [μM]	IC_50_ sEH-NTD^a^ [μM]	IC_50_ sEH-FL (P)^b^ [μM]	IC_50_ sEH-FL (H)^c^ [μM]	IC_50_ sEH-FL (P)^b^ [μM]	IC_50_ sEH-FL (H)^c^ [μM]
**49**	0.059 ± 0.003	1.22 ± 0.08	>30	0.49 ± 0.02	1.9 ± 0.1	2.2 ± 0.5	1.8 ± 0.2	6.7 ± 0.4
**2**	0.058 ± 0.005	4 ± 1	9.5 ± 0.5	n.d	>30	>30	2.8 ± 2.2	>30

a Phosphatase activity of the isolated N-terminal domain.

b Phosphatase activity of the full-length sEH protein.

c Hydrolase activity of the full-length sEH protein. IC_50_ are expressed as the mean ± SD (*n* = 3, triplicates). n.d. not determined.

### In vitro and In Vivo Characterization

We investigated the mode of inhibition of compound **49**. Because our preferred substrate, FDP, contains two cleavable phosphate moieties, it is challenging to accurately determine the enzymatic parameters for sEH using this substrate. Therefore, we employed DiFMUP (6,8-difluoro-4-methylumbelliferyl phosphate), which contains a single phosphate group.^25^ Plotting the DiFMUP concentration against the reaction velocity (Figure 3A) showed that increasing concentrations of compound **49** reduce the *V*_max_ of sEH-FL (P). Consistent with this observation, the IC_50_ value of compound **49** remained unchanged across varying DiFMUP concentrations (Figure 3B), arguing against a competitive mechanism of inhibition. Similarly, the inhibitory potency of compound **49** was unaffected by increasing concentrations of the sEH-NTD cofactor Mg^2+^ (Figure 3C). Finally, variation of the preincubation time did not alter the IC_50_, suggesting that compound 49 does not act via a covalent mechanism (Figure 3D).

**Figure 3:**
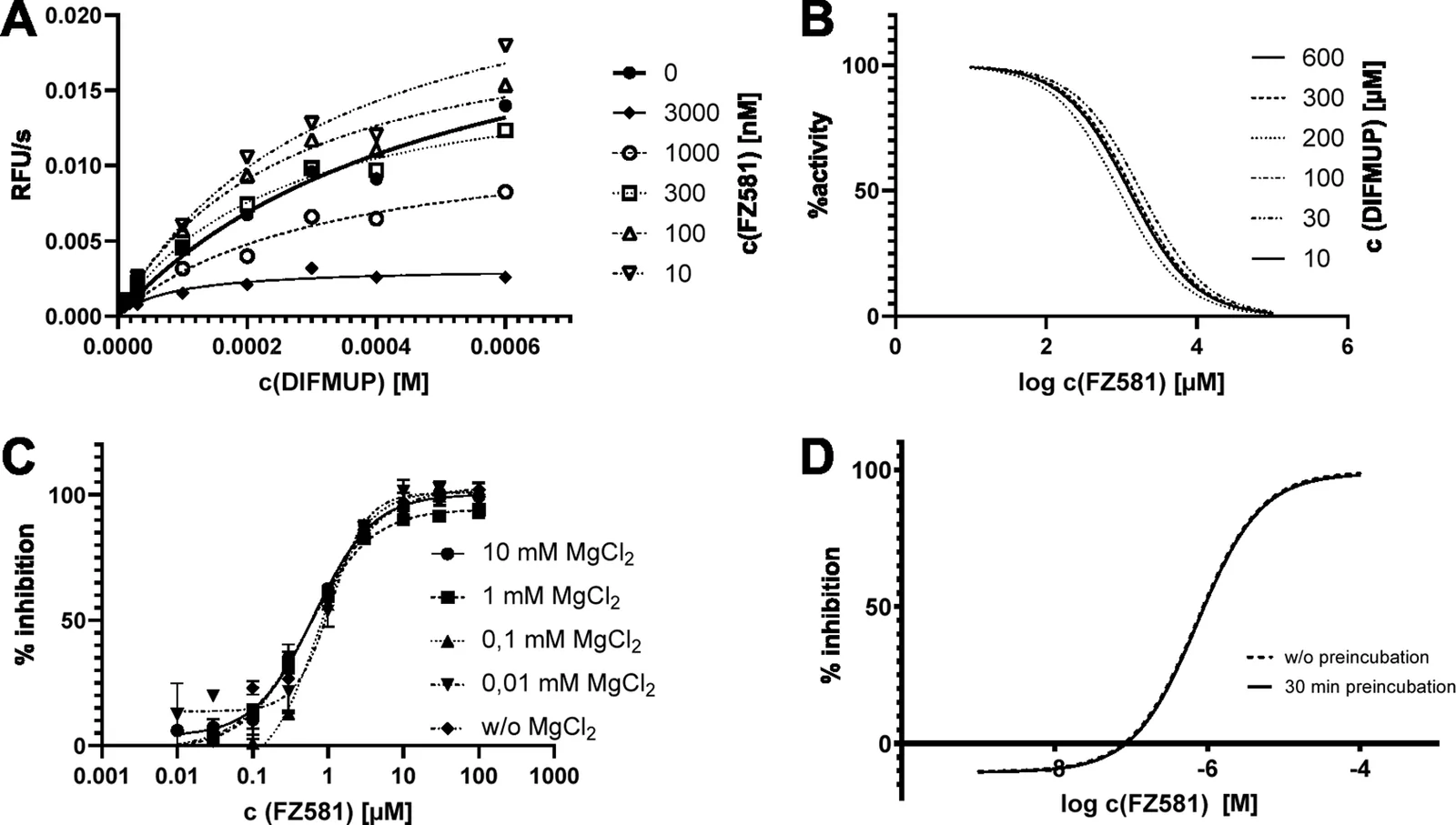
Enzyme inhibition of human sEH-FL (P) (A) Michaelis–Menten plot of DIFMUP conversion with varying concentrations of FZ581 (**49**). (B) Dose-response plot of sEH-FL (P) inhibition with varying concentrations of the fluorogenic substrate DIFMUP. (C) Dose-response plot of sEH-FL (P) inhibition with varying concentrations of the cofactor Mg^2+^. (D) Dose-response plot of sEH-FL (P) inhibition with varying preincubation time.

Following up on the characterization of compound **49** as a potential pharmacological tool, the solubility and microsomal stability were determined next. At neutral pH, the compound possessed a high solubility of over 1000 μM. The sEH phosphatase assay was performed at pH 5.75, and at these conditions, compound **49** reached a solubility limit at 100 μM (Table 6).^21^ Furthermore, compound **49** possessed a low intrinsic clearance (CL_int_) in vitro after incubation in rat liver microsome. We additionally analyzed the metabolic stability of compound **46**, which has the methyl group replaced by a chlorine (Figure S1). **46** was even more stable in vitro (CL_int_ = 0.8 ± 0.3 mg/μL·min), indicating that the methyl group of compound **49** might be a metabolically labile site. However, the altogether good solubility and low intrinsic clearance rendered compound **49** suitable for further characterization.

**Table 6:** Solubility and In Vitro Metabolic Stability of **49**

cmpd	solubility limit at pH 7.00–7.30^a^ (μM)	solubility limit at pH 5.75^b^ (μM)	in vitro CL_int_ ^c^ (mg/μL·min)
**49**	>1000	100	8.2 ± 0.7

a DPBS buffer (*n* = 1, triplicates).

b Assay buffer (*n* = 1, triplicate).

c In vitro CL_int_ (intrinsic clearance) after incubation of compound (10 μM) in rat liver microsome (*n* = 3). Data are expressed as the mean ± SEM.

Next, the off-target profile of compound **49** was assessed (Table 7). Given the structural similarity to several approved fenamic acid derivatives, which are nonsteroidal anti-inflammatory drugs (NSAIDs) inhibiting cyclooxygenase (COX) activity, inhibition of compound **49** against the two isozymes COX-1 and COX-2 was first determined.^1^ However, an over 50-fold higher IC_50_ for COX-1 in comparison to sEH-FL phosphatase and complete inactivity up to a concentration of 100 μM against COX-2 was measured. Then, selectivity against a variety of other phosphatases was investigated by DSF. Incubation of compound **49** with both human and murine sEH NTD furnished Δ*T*_m_ shifts comparable to screening hits **4** and **5** but at a 10-fold lower concentration. It should be noted that DSF is suited for detecting protein-ligand binding interactions but does not reflect the pharmacological action of a molecule, meaning that a thermal stabilization would not necessarily indicate protein function modulation. However, since compound **49** did not exhibit significant binding toward any of the other 9 tested phosphatases, we did not follow up with functional assays and concluded that these results demonstrate the selectivity of the test compound, which is crucial for the deployment as a chemical tool.

**Table 7:** Selectivity Screening of Compound **49**

off-target	IC_50_ [μM]
COX-1^a^	63
COX-2^a^	inactive^b^

off-target^c^	ΔT_m_ [ °C]
human sEH-NTD	3.6
mouse sEH-NTD	3.2
PTPN11	–1.1
PTPRD	–0.1
PTPN14	0.3
PTP4A2	–0.3
PTPN3	–1.0
PTPN7	0.3
Dusp7	–0.9
Dusp12	0.0
Dusp18	0.5

a Off-target activity against human COX isoforms and human phosphatases. Fluorescence-based enzyme activity assay (*n* = 1, duplicates), results presented as IC_50_ [μM].

b Inactive at highest compound concentration measured (100 μM).

c DSF assay was measured with 50 μM compound **49** (*n* = 1, triplicates), results presented as Δ*T*_m_[ °C].

Cellular target engagement is a critical factor in the development of pharmacological tools.^26^ Therefore, we performed a HiBiT-based intact-cell CETSA^27,28^ in HeLa^sEH–HiBiT^ cells^29^ using compound **49**. The melting temperature of sEH in the cellular environment increased by 1.9 and 5.8 °C upon treatment with 1 μM and 3 μM compound **49**, respectively (Figure 4A), demonstrating robust cellular target engagement.

**Figure 4:**
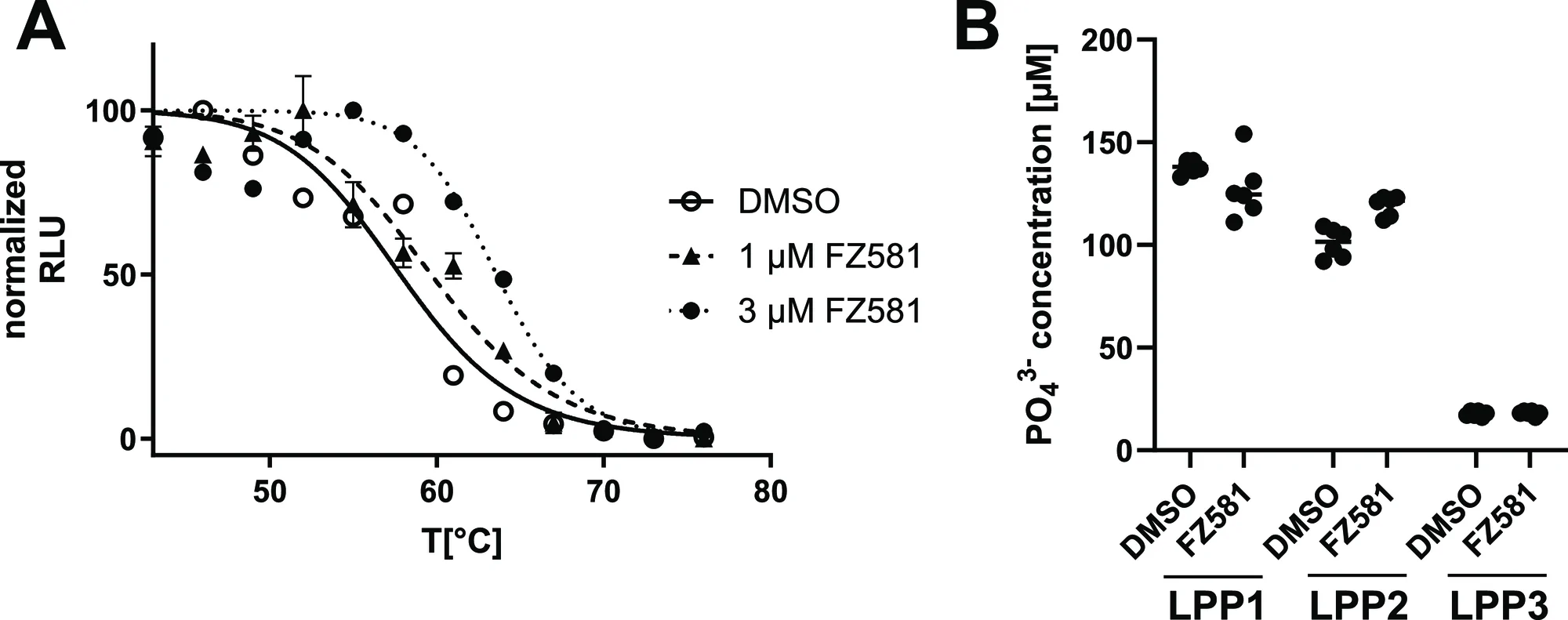
(A) HiBiT-based intact cell CETSA in HeLa^sEH–HiBiT^ cells with compound FZ581 (**49**). (B) Selectivity testing of compound **49** (FZ581) against lipid phosphate phosphatases (LPPs) LPP1–3.

sEH-P is not the only enzyme capable of hydrolyzing LPAs in vivo. LPPs hydrolyze phospholipids in a largely nonspecific manner, including LPAs.^30^ Therefore, it was important to investigate whether compound **49** is selective against LPPs. We found that compound **49** did not affect the enzymatic activity of recombinant LPP1–3 at concentrations up to 100 μM (Figure 4B).

In alignment with **49** exhibiting inhibitory activity across different species in vitro, we investigated the potential of **49** as a pan-species sEH-P inhibitor in vivo. First, we ensured that the conversion of the endogenous sEH-P substrate 1-oleyl LPA (sn1-18:1 LPA) by sEH-FL is also inhibited by **49**. Dose-dependent inhibition of 1-oleyl monoacyl glycerol (sn1-18:1 MAG) formation through inhibition of recombinant mouse sEH was observed (Figure 5A). Pharmacokinetic and pharmacodynamic studies were then conducted in mice and rats.

**Figure 5:**
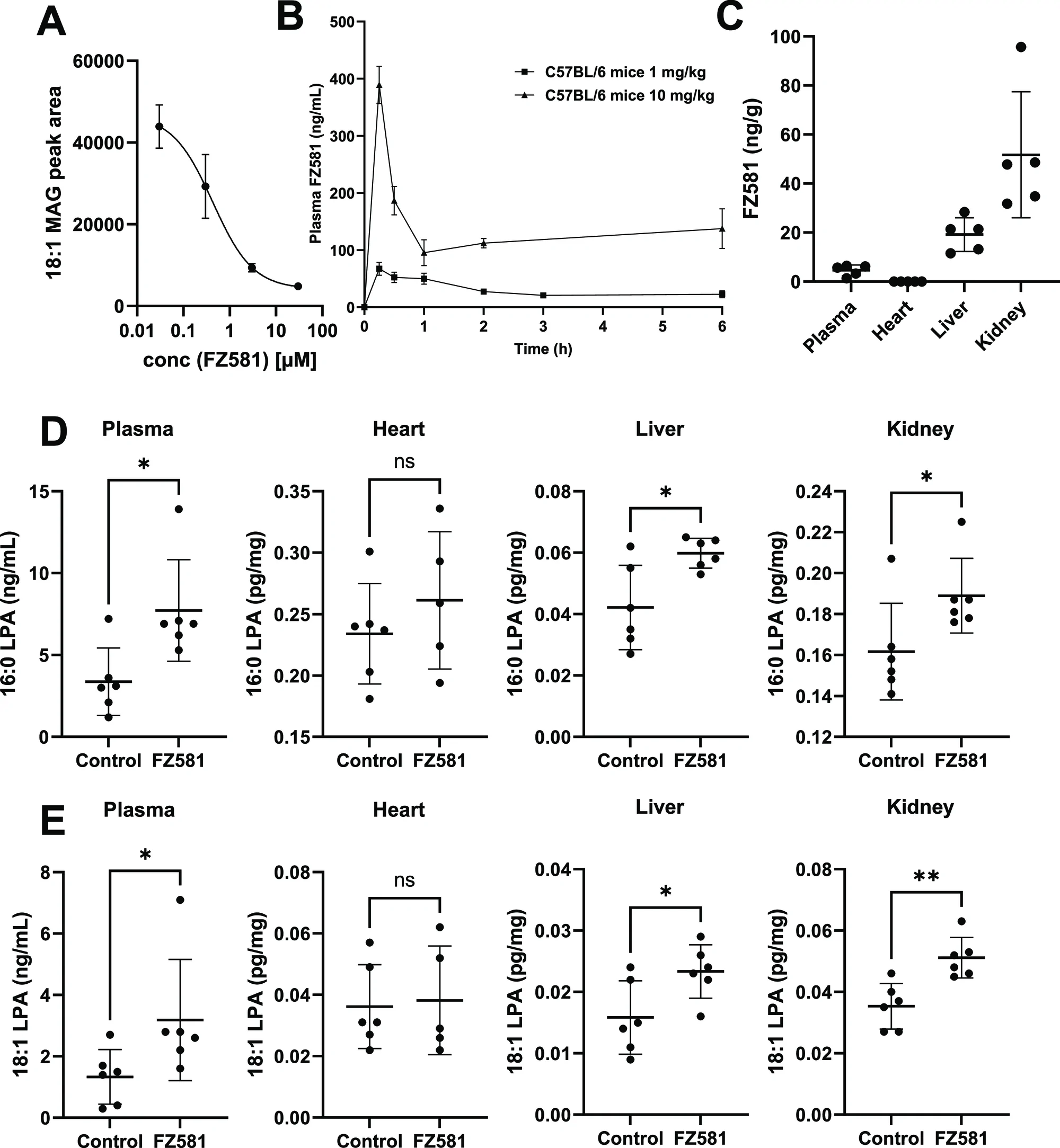
(A) Levels of 1-oleyl monoacyl glycerol (sn1-18:1 MAG) formed from 1-oleyl lysophosphatidic acid (sn1 18:1 LPA) by recombinant mouse sEH in the presence of increasing concentrations of FZ581 (**49**). Data are given as mean ± SEM. (B) Evolution of plasma levels of FZ581 (**49**) in C57BL/6 mice during 6 h after oral administration at 1 mg/kg (*n* = 7). Data are given as mean ± SEM. (C) Tissue levels of FZ581 (**49**) in C57BL/6 mice 2 h after oral administration at 1 mg/kg (*n* = 5). (D) Tissue levels of 1-palmitoyl LPA (sn1-16:0 LPA) in C57BL/6 mice 2 h after oral administration at 1 mg/kg (*n* = 6) vs vehicle (*n* = 6). (E) Tissue levels of 1-oleyl LPA (sn1-18:1 LPA) in C57BL/6 mice 2 h after oral administration at 1 mg/kg (*n* = 6) vs vehicle (*n* = 6). Data are given as mean ± SEM. Differences between groups were evaluated using Mann–Whitney tests. *P < 0.05.

To investigate the pharmacokinetics of compound **49** in mice, 1 and 10 mg/kg were administered p.o. to male C57BL/6 mice. Dose-dependent pharmacokinetics were observed (Table S2), but their interpretation is hampered due to the appearance of a second plasma peak in some animals, suggesting a possible reabsorption phenomenon. Monitoring of compound **49** after intravenous administration at 0.1 mg in mice (Figure S2) allowed the calculation of an oral bioavailability of 16.9 ± 5.4% for the dose of 1 mg/kg p.o. and 8.2 ± 2.5% for the dose of 10 mg/kg p.o (Figure 5B). However, the application of 1 mg/kg and 10 mg/kg of **49** led to significant, but not dose-dependent, increase in LPAs after 6 h (Figure S3). Then, we quantified the tissue-specific concentrations of **49** (Figure 5C) and LPAs (Figures 5D,E). sEH is known to be highly expressed in heart, liver, and kidneys.^31^ After administration of 1 mg/kg of compound **49**, we could observe after 2 h that the concentrations of **49** in liver and kidney were much higher than plasma concentrations, while we could not detect any compound **49** in the heart. Notably, levels of 16:0 LPA and 18:1 LPA were significantly increased in plasma, liver, and kidney, but not in heart compared to the vehicle-treated mice.

Then, 1 mg/kg was administered p.o. to male Sprague–Dawley rats, and the evolution of the plasma concentration of compound **49** was again monitored over 6 h (Figure 6A). A summary of the main noncompartmental analysis parameters is given in Table S2. As observed in mice, there was an increase from the baseline in the plasma levels of LPAs (Figure 6B). In addition, we can demonstrate that the plasma levels of 3 epoxy fatty acids known to be metabolized by sEH-H were not different 6 h after vehicle or administration of **49** (Figure 6C).

**Figure 6:**
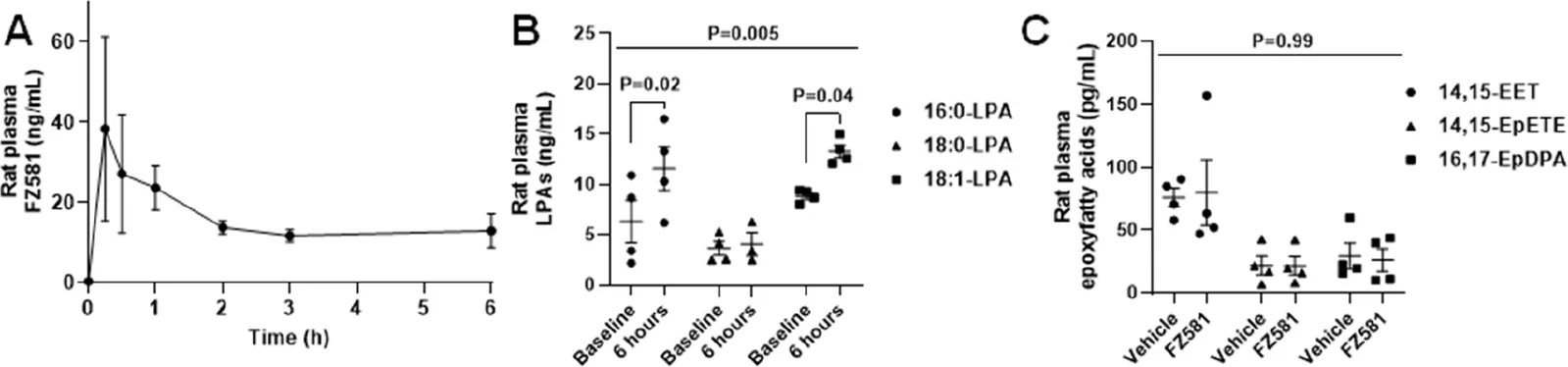
(A) Evolution of plasma levels of FZ581 (**49**) after oral administration in Sprague–Dawley (SD) rats at 1 mg/kg (*n* = 6) for 6 h. (B) Evolution of plasma levels of 1-palmitoyl LPA (sn1-16:0 LPA), 1-steraoyl LPA (sn1-18:0 LPA), and 1-oleyl LPA (sn1-18:1 LPA) induced by FZ581 (**49**) at 1 mg/kg in rats after 6 h (*n* = 4). (C) Plasma levels of the epoxy fatty acids 14,15-epoxyeicosatrienoic acid (14,15-EET), 14,15-epoxyeicosatetraenoic acid (14,15-EpETE), and 16,17-epoxydocosapentaneoic acid (16,17-EpDPA) in rats 6 h after administration of vehicle or FZ581 (**49**) at 1 mg/kg (*n* = 4 per group).

## Conclusions

sEH is a bifunctional enzyme with a C-terminal hydrolase activity and a N-terminal phosphatase activity. While the role of the hydrolase activity is well studied and its therapeutic potential recognized, the physiological role of the phosphatase domain is less known. Recent in vivo studies indicate that inhibition of sEH-P could potentially represent a pharmacotherapeutic strategy to treat obesity and cardiac ischemic complications.^32^ Development of a pharmacological inhibitor could enable the validation as a drug target. However, previous efforts to develop selective sEH-P inhibitors were challenged by varying activities between different species isoforms and lack of selectivity toward sEH-H. DSF screening of a 480-membered drug-derived fragment library against both human and mouse sEH-P resulted in the identification of fenamic acids as inhibitors of both isoforms with good ligand efficiencies. Optimization of this scaffold led to the discovery of **49**, an inhibitor with IC_50_ in the single digit μM range against human, mouse, and rat isoforms. Compound **49** exhibits high aqueous solubility, low intrinsic clearance, and good preliminary off-target selectivity against COX despite the prevalence of the scaffold in approved NSAIDs, as well as several lipid and protein phosphatases. Moreover, initial in vivo PK/PD studies have been performed to evaluate compound **49** as a tool compound to study the therapeutic potential of sEH-P in vivo. Upon oral administration of **49**, only plasma levels of the main sEH-P substrates significantly increased, while plasma levels of three sEH-H substrates were not affected. Further in vivo studies are necessary to investigate dose-dependency of the PD effects upon administration of compound **49**. The cross-talk between the substrates and the enzymatic activities of sEH-H and sEH-P has been recently investigated in vitro,^23^ however, its implications in in vivo settings remain currently unclear. The development of **49** as the first pan-species sEH-P inhibitor will facilitate future studies on the physiological and pathophysiological role of sEH-P. Furthermore, 49 represents a novel chemotype of sEH-P inhibitors which could potentially serve as a starting point for development of sEH degraders.^29,33–35^

## Experimental Section

### In vitro Assays

#### Cloning of the Murine sEH NTD

The cloning of the murine sEH NTD was performed similar to the full-length construct described by Lillich et al.^36^ but by using the following primers: for: ATATATGCTCTTCTAGTGCGCTGCGTGTAGC rev: TATATAGCTCTTCATGCGGCCTCAGGAAACTGTGTC.

In brief, the murine sEH-PD (aa2-224) with an N-terminal 10xHis-Tag construct was generated via PCR and FX-cloning.^37^ The murine sEH-PD insert was generated by PCR using DNA provided by the research group of Prof. Dr. Hammock as a template and the described primers. After amplification, the PCR mixture was separated via an agarose gel and the inset was extracted using the GeneJET Gel Extraction Kit (Thermo Scientific) according to supplier’s protocol. Both the inset and the pINIT_cat vector were digested with SapI (New England BioLabs Inc.) and after heat inactivation ligated using the T4 DNA ligase (New EnglandBioLabs Inc.). The mixture was used to transform chemically competent *Escherichia coli* MC1061 cells via standard heat shock protocol and plated on agarose plates containing 35 μg/mL chloramphenicol. After ∼12 h, single clones were picked and used to inoculate 6 mL of LB culture, supplemented with 35 μg/mL chloramphenicol. After 12 h, the cells were spun down and the plasmids were extracted using the GeneJET plasmid miniprep kit (Thermo Scientific) according to supplier’s protocol. The purified murine sEH-PD-pINIT_cat vector was mixed with p7XNH3 vector, digested with SapI, and after heat inactivation, ligated using the T4 DNA ligase. The ligation mixture was subsequently used to transform chemically competent *E. coli* MC1061 cells with a standard heat shock protocol and plated on agarose plates, containing 100 μg/mL kanamycin. After 12 h, single clones were picked and used to inoculate 6 mL of LB culture, supplemented with 100 μg/mL kanamycin. After 12 h, plasmids were extracted using the GeneJET plasmid miniprep kit (Thermo Scientific) according to supplier’s protocol. The msEH-PD sequence was verified via Sanger sequencing (Microsynth Seqlab) using standard primers T7 and T7term. The final construct carried an N- terminal 10xHis-tag, followed by a 3C protease cleavage site and, a serine, the msEH-PD from aa2-aa224 with an additional alanine. For DNA and protein sequence of msEH-P-p7XNH3 (10xHis-Tag_3C-protease-cleavage-site_msEH-P), refer to the Supporting Information. The msEH-P-p7XNH3 vector was used to transfect *E. coli* Rosetta (DE3) cells with a standard heat shock protocol and then used to inoculate 30 mL of LB media containing 35 μg/mL chloramphenicol and 100 μg/mL kanamycin. After ∼12 h, 10 mL were used to inoculate 1 L of LB media containing 35 μg/mL chloramphenicol and 100 μg/mL kanamycin. The expression and purification were performed as described for the human sEH-PD by Kramer et al.^16^ The purified sEH-batch used in this work originates from the work by Ehrler et al.^38^

#### Other Constructs

The human sEH-NTD published by Kramer et al.^16^ was used for the activity assay and DSF screening. The full-length murine sEH was previously published by Lillich et al.^36^ The full-length human sEH was previously published by Lukin et al.^39^

#### Protein Expression and Purification

The human sEH-PD used for the DSF screening and the activity assay, as well as the mouse and human sEH full length used in the performed activity assays were expressed and purified as published previously by Klingler et al., Lillich et al., and Lukin et al.^21,36,39^ The proteins for the assay were flash-frozen in liquid nitrogen after the addition of 20% v/v glycerol and stored at −80 °C.

#### DSF Screening of Prestwick Fragment Library

DSF was performed as published previously by Kramer et al.^16^ using frozen human or mouse sEH-PD. It was used as primary screening method for the Prestwick fragment library and all experiments were performed as *n* = 2. In brief, the screening was performed in MicroAmp fast 96-well plates and 0.8 μL of either 25 mM Preswick drug-derived fragment library dilution in DMSO (final concentration of 500 μM) or pure DMSO (as blank) were placed in the respective wells. Then 39.2 μL of the master mix, containing the protein and SYPRO in the appropriate buffer was added to every well. The final concentrations were 5 μM sEH-PD (human or mouse), 43.18 mM sodium acetate, 8.64 mM MgCl_2_, 0.64% v/v glycerol, 0.01% w/v Triton X-100, 2.0005% DMSO, and 2.5x SYPRO (pH 5.75). The measurements were performed using a iCycler iQ single-color real time PCR device (BioRad) with a heat rate of 1 °C per min, an excitation wavelength of 490 nm, and an emission wavelength of 570 nm. The first derivative curve given by MyIQ 1.0 software was analyzed using Microsoft Excel 2013 by comparing the maxima. A shift more than 1 °C compared to the DMSO control was assumed as potentially binding of the fragment.

For the selectivity screening against other phosphatases, compound **49** was added at a final concentration of 50 μM. For the sEH-PD, the same buffer was used but other phosphatases were measured at neutral pH, as they did not tolerate pH 5.75 and vice versa.

#### FDP-Based Phosphatase Assay

The FDP assay was performed as published previously by Klingler et al.^21^ using frozen sEH-PD as well as full-length human, mouse, and rat sEH. In brief, the assay was performed in a black 96-well microplate in which 1 μL of compound in DMSO (or DMSO only in the case of the positive control) was mixed with 89 μL of protein solution (final concentration of 0.1 μM for sEH-PD, full-length human sEH, and full-length rat sEH and 0.01 μM for full-length mouse sEH) in phosphatase assay buffer (50 mM acetate, 10 mM MgCl_2_, pH 5.75) supplemented with 0.01% w/v Triton X-100. For the blank, assay buffer without protein was used. The plates were incubated at rt for 30 min to allow binding of the inhibitor. To start the reaction, 10 μL of FDP solution (10 μM final concentration) in assay buffer was added, and the fluorescence changes (λ_ex_ = 485 nm, λ_em_ = 525 nm) were detected at 30 time points over the duration of 30 min using an Infinite F200 PRO plate reader (Tecan, Crailsheim, Germany). All samples were measured in triplicate. The percent inhibition was calculated relative to the activities of the blank well (without protein) and positive control well (without inhibitor). To calculate IC_50_ values, data obtained from measurements with at least six different inhibitor concentrations were fitted with a sigmoidal dose-response function using GraphPad Prism software (version 5.03; GraphPad Software, Inc.).

#### Inhibition of LPP1–3 by SWE101 and FZ581 (**49**)

LPP1–3 proteins were purified as described previously.^40^ Inhibitors SWE101 and FZ581 (**49**) were freshly dissolved in DMSO to 10 mM stock solutions. LPA was dissolved in chloroform/methanol (3:1, v/v), dried under nitrogen, and resuspended in 1% Triton X-100 to prepare a 10 mM stock. Enzyme reactions were performed in buffer containing 50 mM Tris–HCl (pH 7.5) and 0.51% Triton X-100 (final concentration). For inhibition assays, 0.9 μL (final concentration of 100 μM) inhibitor and 1 μL of 0.03 mg mL^–1^ protein were preincubated at RT for 10 min prior to initiation with 0.9 μL LPA. The total reaction volume was 90 μL, and reactions were carried out at 37 °C for 10 min. Subsequently, 75 μL of reaction mixture was mixed with 25 μL freshly prepared malachite green development solution (10 mL 2.6 mM malachite green, 2.5 mL 7.5% ammonium molybdate, 0.2 mL 100% Tween 20) and incubated for 10 min at RT. The reaction was terminated with 10 μL 5% oxalic acid and incubated for 30 min in the dark before measuring absorbance at 630 nm. An enzyme-free control was included for background subtraction. Data were analyzed using GraphPad Prism 8.0.2.

#### HiBiT-Based Intact Cell CETSA

In order to determine the melting point of sEH in intact cells, a CETSA was conducted. The assay was performed using HeLasEH-HiBiT cells29 and the slightly modified protocol that had been published by the EUbOPEN consortium (https://www.eubopen.org/sites/www.eubopen.org/files/attachments/2023/CETSAprotocolAug152023.pdf).27 HeLasEH-HiBiT cells were cultivated in DMEM (Thermo Fisher Scientific, #41965-039) supplemented with 10% Corning fetal bovine serum (Corning, 35-079-CV), penicillin (100 units/ml), streptomycin (100 μg/mL) (Gibco #15140), and 1 mM sodium pyruvate (Gibco #11360) at 37 °C and 5% CO_2_. On the day of the CETSA, cells were harvested using Gibco trypsin–EDTA and suspended in Opti-MEM I reduced serum medium (ThermoFischer, 11058021). In a 96-well PCR plate (Applied Biosystems by Thermo Fisher Scientific, 4346907), 0.2 × 10^6^ cells/ml in Opti-MEM I reduced serum medium were mixed with the indicated compound concentration in DMSO or pure DMSO as reference followed by centrifugation for 1 min at 250 rpm. The plates were sealed with an AeraSeal film (SIGMA-Aldrich, A9224-50EA) and incubated for 1 h at 37 °C and 5% CO_2_. Afterward, the seal was replaced by a PCR-clear seal (Sarstedt, 95.1994) and incubated for 1 min at 22 °C and 3 min at desired temperature gradient (43 °C–76 °C, 3 °C interval), followed by a cooling step to chill samples to 22 °C on a Veriti 96-well thermal cycler. The top of the plate was cooled down with an ice block for 30 s. 10 μL of the cell suspension were transferred into white 384-well plates (Greiner, 784075) and mixed with 10 μL Nano-Glo HiBiT lytic solution (Promega, #N3030), prepared according manufacturer’s protocol, and incubated for 10 min on an orbital shaker in the dark. Luminescence was measured at a Tecan SPARK plate reader with a settle time of 100 ms and an integration time of 450 ms. The data was then graphed using GraphPad 7.05, and the cellular melting points (Δ*T*_m_) were calculated as IC50 using the nonlinear four parameter curve fit.

#### PHOME-Based Hydrolase Assay

The fluorescence-based sEH hydrolase activity assay was performed as described previously in a 96-well format with some minor modifications.^41^ In short, full-length sEH (3 nM final concentration for all tested proteins) was dissolved in hydrolase assay buffer (Bis-Tris buffer, pH 7) supplemented with 0.1 mg mL^–1^ BSA as well as Triton-X 100 to a final concentration of 0.01%. Protein solution (89 μL) was incubated with 1 μL of different concentrations of compounds (final DMSO concentration 1%) for 30 min at rt. The reaction was started by addition of 10 μL of a cyano­(6-methoxy-2-naphthalenyl)­methyl 3-phenyloxiran-2-ylacetate (PHOME) solution (final concentration of 50 μM). Hydrolysis of the nonfluorescent substrate by sEH to release fluorescent 6-methoxynaphthaldehyde was measured (λ_ex_ = 330 nm, λ_em_ = 465 nm) using a Tecan Infinite F200PRO plate reader. The reaction was monitored over a duration of 45 min (one point every minute). A blank control (no protein and no compound) and a positive control (no compound) were carried out as well. All measurements were performed in triplicate.

#### Determination of the Water Solubility Limit

In a transparent 96-well flat-bottomed microtiter plate, 1 μL of a DMSO compound solution with a defined concentration (concentration series in DMSO) was placed per well. For the background measurement, 1 μL of pure DMSO was used. To each of these, 99 μL of DPBS buffer solution (Gibco, pH 7.0–7.3) was added. The final DMSO concentration in this assay was 1% v/v. The plate was transferred to an Infinite M200 plate reader (Tecan), shaken for 10 s with an amplitude of 1 mm, and then the absorbance was measured at 650 nm. All dilutions were performed as triplicates. In the evaluation, the mean value of the background measurement was compared with the respective mean value of each dilution. A compound was defined as failed (no longer soluble) if the difference between the two measurements was greater than 0.0005. The concentration of the highest dilution at which the mean value did not yet deviate from the background was then defined as the maximum solubility of the compound.

#### Metabolic Stability Assay in Liver Microsomes

The solubilized test compound (5 μL, final concentration 10 μM) was preincubated at 37 °C in 432 μL of phosphate buffer (0.1 M, pH = 7.4) together with 50 μL of NADPH regenerating system (30 mM glucose-6-phosphate, 4 U/mL glucose 6-phosphate dehydrogenase, 10 mM NADP, 30 mM MgCl_2_). After 5 min, the reaction was started by the addition of 13 μL of microsome mix from the liver of Sprague–Dawley rats (Invitrogen; 20 mg protein/mL in 0.1 M phosphate buffer) in a shaking water bath at 37 °C. The reaction was stopped by adding 500 μL of ice-cold methanol at 0, 15, 30, and 60 min. The samples were centrifuged at 5000*g* for 5 min at 4 °C, and the test compound was quantified from the supernatants by HPLC: the composition of the mobile phase was adapted to the test compound in a range of MeOH 40–90% and water (0.1% formic acid) 10–60%. Flow rate: 1 mL/min; stationary phase: Purospher STAR, RP18, 5 μm, 125 × 4; precolumn: Purospher STAR, RP18, 5 μm, 4 × 4; detection wavelength: 280 nm; injection volume: 50 μL. Control samples were performed to check the test compound’s stability in the reaction mixture. First control was without NADPH, which is needed for the enzymatic activity of the microsomes, second control was with inactivated microsomes (incubated for 20 min at 90 °C), and third control was without the test compound (to determine the baseline). The amounts of the test compound were quantified by an external calibration curve. The in vitro intrinsic clearance was calculated by eq 2, wherein k represents the -gradient of the ln peak area ratio plotted against time.

2 $$Intrinsic\;clearance\;\left({CL}_{\mathrm{int}}\right)\;\left[\frac{\mu L}{\min\cdot mg}\right]=\left(\frac{incubation\;volume\;\left[\mu L\right]}{protein\;in\;the\;incubation\;\left[mg\right]}\right)\times \frac{k}{\left[\min\right]}$$

#### COX Activity Assay

The COX activity assay was performed by BPS Bioscience (San Diego, US). In brief, a 100 mM DMSO stock solution of compound **49** was diluted to 10X concentration in 10% DMSO in assay buffer and 10 μL of the dilution was added to a 100 μL reaction so that the final concentration of DMSO from the compounds is 1% in all reactions. All reactions were conducted at RT. The reaction mixture contains 60 μL of 1X COX Assay Buffer, 10 μL of respective enzyme, 10 μL of compound **49**, 10 μL of 1 mM Ampliflu Red, and 10 μL of 0.5 mM arachidonic acid. The test compounds were incubated for 5 min before addition of the detection reagent (Ampliflu Red, 100 μM final concentration) and the substrate arachidonic acid (50 μM final concentration). The reaction mixture was incubated for 5 min (COX1) and 10 min (COX2) before measuring fluorescence (ex. 535 nm, em. 590 nm). For the negative control, assay buffer without COX enzyme was used. Fluorescence signals were measured using a Tecan Infinite M1000 plate reader.

#### In vitro Conversion of 18:1 LPA

FZ581 (**49**) was tested at final concentrations of 30 μM, 3 μM, 0.3 μM, and 0.03 μM, respectively. 1 μL of **49** (in DMSO) was added to a mixture of 18:1-olyolyl-lysophosphatic-acid (18:1-LPA) substrate ([*S*]_final_ = 5 μM) and purified recombinant mouse full-length sEH ([*E*]_final_: 10 nM) in Bis/Tris buffer (25 mM; pH:7.0, 1 mM MgCl_2_ and 0.1 mg mL^–1^ BSA) to final volume of 100 μL. The reaction mixture was incubated at RT for 30 min. The reactions were then quenched by adding 100 μL of pure acetonitrile. The analysis of the formed 1-oleoyl-glycerol product was carried out on Acquity UPLC (Waters, Eschborn, Germany) coupled to a single quadrupole mass detector (QDa, Waters). LC separation was carried out on a Supelco Discovery “Bio Wide Pore” C-5 RP precolumn (Sigma-Aldrich, St Louis, MO) with the following dimensions: 20 mm × 2.1 mm, particle size 3 μm, and pore size of 30 nm. The injection volume was 5 μL and the analyte was eluted with a gradient composed of water containing 0.1% acetic acid as solvent A and acetonitrile as solvent B at a flow rate of 0.5 mL/min applying the following gradient: 0–0.50 min isocratic 45% B, 0.50–2.0 min linear from 45% to 60% B, 2.0–2.10 min linear from 60% to 95% B, 2.10–3.10. min linear 95% B, 3.10–3.20 min linear from 95% to 45% B, and 3.20–5.0 min reconditioning at 45% B. The enzymatically formed product (1-oleoyl-glycerol) was analyzed in the positive single ion mode [M + H^+^]^+^ using the Selected Ion Monitoring (SIM) approach and monitoring (*m*/*z* = 357.5).

### In vivo Studies

#### Animals

Adult, 12 week-old male Sprague–Dawley rats and adult, 6 week-old male C57BL/6 were purchased at Janvier Laboratories (Le Genest-Saint-Isle, France) with an average weight at delivery of 350 and 15 g, respectively. They were housed in a temperature-controlled room (20 to 24 °C) and maintained in a 12 h light/12 h dark cycle. Food and water were provided ad libitum. All experimental procedures were approved by and conducted in accordance with the standards and ethical rules (CENOMEXA #24107).

The tissue-specific quantification of **49** after p.o. administration (1 mg/kg) was performed by Enamine (Kyiv, Ukraine). Study design, animal selection, handling, and treatment were all in accordance with the Enamine PK study protocols and Institutional Animal Care and Use Guidelines (BACUC approval number #GUF-PK-29012024). Animal treatment and sample preparation were conducted by the Animal Laboratory personnel at Enamine/Bienta. Male C57BL/6J mice (9 weeks old, body weight ranging from 21.1 to 24.5 g and average body weight across all groups of 22.5 g, SD = 1.2 g) were used in this study.

#### Surgery

Animals were anaesthetized with xylazine (10 mg/kg) followed by 1–2% isoflurane, and the right jugular vein was cannulated with silicone tubing. The catheter was tunneled subcutaneously, exteriorized in the neck region, and connected to a vascular access harness. For analgesia, rats were treated twice with buprenorphine (0.05 mg/kg s.c.) directly after induction of anesthesia for catheter surgery. Catheters were rinsed with 50 μL heparin sodium solution (500 IU/mL). Prior to the first blood sampling, animals were connected to a tether system that allowed for blood sampling in the freely moving rat.

#### Treatment and Sample Collection

Compound **49** at the dose of 1 mg/kg and 10 mg/kg and vehicle (1% DMSO, water for injection) were administered orally by gavage in mice and/or rats. In addition, compound **49** was administered intravenously at the dose of 0.1 mg/kg in mice. Serial blood sampling time points from each rat and mouse in treatment group 1:0.25, 0.5, 1, 2, 4, and 6 h post dose were drawn. Blood was collected from the vascular access harness in iced tubes containing EDTA. Terminal blood was retrieved by exsanguination under isoflurane anesthesia. EDTA-whole blood samples were centrifuged (10 min at 3000*g*, 4 °C) and plasma was collected within 15 min.

#### Quantification of **49**

Stock solution of compound **49** at 0.434 mg mL^–1^ in DMSO was diluted to obtain a working stock solution (A1) in methanol (MeOH) to a final concentration of 200 μg/mL. Secondary working stock solution (A2) was prepared by a half dilution. A2 was subsequently freshly diluted for the preparation of calibration standards to a final concentration in the range of 1–1000 ng/mL in plasma with coefficient of determination *r* ^2^ >0.99. Due to the absence of isotope-labeled internal standard (IS) for compound **49**, quantification was performed by external calibration using peak area. Plasma levels of compound **49** were determined by ultrahigh performance liquid chromatography coupled with tandem mass spectrometry (UHPLC–MS/MS) after protein precipitation. Briefly, 50 μL of plasma was deproteinized with 100 μL of MeOH. Each sample was vortexed and centrifuged (5 min, 16.100*g*). Supernatant was then transferred into an autosampler vial and 10 μL was injected into the UHPLC–MS/MS system.

#### Tissue-specific Quantification of **49**, LPAs, and Epoxy Fatty Acids

Animals were randomly assigned to the treatment or control group before the pharmacokinetic study (6 each). All animals were fasted for 4 h before dosing (p.o. route of administration, formulation DMSO/water 1:99). One sampling time point at 120 min was set for this pharmacokinetic study. Mice were injected i.p. with 2,2,2-tribromoethanol at the dose of 150 mg/kg prior to drawing the blood. Blood collection was performed from the orbital sinus in microtainers containing K_3_EDTA. Animals were sacrificed by cervical dislocation after the blood sample collections. Blood samples were centrifuged for 10 min at 3000 rpm. One liver lobe (left lobe), one kidney (left), the left heart ventricle, and half of plasma samples (∼100 μL) were frozen and stored at −70°C until quantification of LPA as described below. The other half of plasma samples (∼100 μL), liver (fragment of right lobe), heart (right ventricle with atrium), and kidney (right) were transferred into 1.5 mL tubes (safe-lock), flash-frozen, and stored at −70 °C until subsequent LC–MS/MS analysis. To this end, a solution of diclofenac (400 ng/mL in water–methanol mixture 1:9, v/v) was used as an internal standard (**IS­(90)**) for quantification of **49** in plasma samples. Plasma samples (40 μL) were mixed with 200 μL of **IS­(90)** solution. After mixing by pipetting and centrifuging for 4 min at 6000 rpm, 3 μL of each supernatant was injected into a LC–MS/MS system. A solution of diclofenac (400 ng/mL in water–methanol mixture 1:4, v/v) was used as an internal standard (**IS­(80)**) for quantification of FZ581 in tissue samples. Tissue samples (weight 42 mg–158 mg) were homogenized with 5 volumes of **IS­(80)** solution (100 mg + 500 μL) using glass beads (115 mg ± 5 mg) in the bullet blender homogenizer for 30 s at speed 8. After this, the samples were centrifuged for 4 min at 14,000 rpm, and 1 μL of each supernatant was injected into LC–MS/MS system. The concentration of **49** in samples was determined using high performance liquid chromatography/tandem mass spectrometry (HPLC–MS/MS). Shimadzu HPLC system comprised 2 isocratic pumps LC-20AD, an autosampler SIL-20ACXR, a subcontroller FCV-14AH, and a degasser DGU-14A. Mass spectrometric analysis was performed using an API 3000 (triple-quadrupole) instrument from AB Sciex (Canada) with an electro-spray (ESI) interface. The data acquisition and system control were performed using Analyst 1.6.3 software from AB Sciex.

#### Determination of Rat Plasma Levels of LPAs and Epoxy Fatty Acids

Epoxy fatty acids and LPAs were purchased from Cayman Chemicals (Ann Arbor, MI, USA) and Avanti Polar Lipids (Alabaster, AL, USA), respectively.

#### Determination of LPAs

Plasma levels of the sEH-P substrates 1-palmitoyl lysophosphatidic acid (sn1-16:0 LPA), 1-steraoyl LPA (sn1-18:0 LPA), and 1-oleyl LPA (sn1-18:1 LPA) were determined by UHPLC–MS/MS. Solution stocks containing 16:0, 18:0, and 18:1 LPA in MeOH were prepared at the following concentrations: 0, 1.5, 3, 15, 30, 75, 150 ng/mL. Calibration curves were obtained by spiking the solution stocks to obtain increasing concentrations (0, 0.5, 1, 5, 10, 25, 50 ng/mL) in phosphate-buffered saline (PBS) with a fixed concentration of the IS (1-heptadecenoyl LPA [*sn-1* 17:1 LPA]). Each analyte presented coefficient of determination *r* ^2^ >0.99, after protein precipitation and lipids extraction. Briefly, 300 μL of plasma were spiked with 20 μL of IS in MeOH. Protein precipitation was performed adding 980 μL of MeOH. Samples were vortexed and centrifuged (5 min, 16.100*g*). Then, liquid–liquid extraction was performed as described previously.^42^ Supernatant was mixed with 100 μL of HCOOH (10%), 2 mL of DCM, and 350 μL of H_2_O. The bottom organic phase was collected after being centrifugated (4.500*g*, 10 min, +4 °C), evaporated under a nitrogen, and reconstituted in 50 μL of MeOH. Due to low plasma volume for baseline sampling times in mice, LPA quantification was performed along with compounds **49** using a simple deproteinization step without IS. Sample was transferred into an autosampler vial and 10 μL was injected into the UHPLC–MS/MS system.

LPAs and compound **49** assays were performed on a UHPLC–MS/MS system consisting of the following Shimadzu modules (Shimadzu Corporation, Marne-la-Vallée, France): a binary pump consisting of coupling two isocratic pumps Nexera LC30AD, an automated sampler SIL-30AC, a column oven CTO-20AC, and a triple-quadrupole mass spectrometer LCMS-8060 operating in the negative ion mode. Chromatographic separation was performed on a Thermo Accucore XL C18 (4 μm particle size, 150 mm length × 3 mm inner diameter; Thermo Fischer Scientific, Waltham, MA, USA). The autosampler temperature was set at 8 °C, the column oven at 50 °C, the injected volume was 10 μL, and the flow rate was 500 μL/min. The mobile phase was 1% acetic acid and 10 mM ammonium formate in water (solvent A) and water (solvent B). Elution was started with 70% A (0–0.5 min), 70–95% A (0.5–5.5 min), 95% A (5.5–7.9 min), 95–70% A (7.9–8 min), 70% A (8–10 min). The source interface parameters and common settings were as follows: interface voltage: −3 kV for compound **49** and −0.5 kV for LPAs; nebulizing gas flow: 3 L/min; heating gas flow: 10 L/min; drying gas flow: 10 L/min; interface temperature: 400 °C; DL (desolvation line) temperature: 250 °C; heat block temperature: 500 °C; and collision gas pressure: 270 kPa. Detection and quantification were performed by scheduled- multiple reaction monitoring (MRM) using a pause time of 3 ms and individual dwell times to achieve sufficient points per peak. We used 1-heptadecenoyl LPA (*sn*-1 17:1 LPA) as IS. Mass transitions (*m*/*z*) and mass spectrometer parameters are summarized in Table S1. All subspecies were analyzed in negative ion mode.

#### Determination of Epoxy Fatty Acids

Plasma levels of the sEH-H substrates 14,15-epoxyeicosatrienoic acid (14,15-EET), 14,15-epoxyeicosatetraenoic acid (14,15-EpETE), and 16,17-epoxydocosapentaneoic acid (16,17-EpDPA) were determined by LC–MS/MS after protein precipitation and solid phase extraction as previously described.^12,13^ Calibration curves were obtained by spiking the standards at the following concentrations: 0, 10, 20, 50, 100, 200, 500, 1000, and 2000 pg/mL in PBS with an identical concentration of the IS (14,15-EET-*d*_11_) at 30 ng/mL. Each analyte presented a coefficient of determination *r* ^2^ >0.99.

Briefly, in LoBind Eppendorf (2 mL), 10 μL of IS in MeOH at 30 ng/mL was added to 500 μL of plasma. After the deproteinizing step by adding 990 μL of MeOH, sample was vortexed and centrifuged (5 min, 16.100*g*). The supernatant was loaded into preconditioned Oasis HLB-SPE 3 cm^3^ 60 mg column. Then, the column was washed by 6 mL of MeOH/water (5/95, v/v) and dried for 20 min. Free epoxy fatty acids were eluted into glass tubes using 0.5 mL of MeOH and 1.5 mL of EtOAc. Eluted compounds were evaporated under nitrogen at RT. Residue was reconstituted by adding 50 μL of MeOH and vortexing. Sample was transferred into an autosampler vial and 10 μL was injected into the UHPLC–MS/MS system.

Quantification of epoxy fatty acids was performed on a UHPLC–MS/MS system consisting of the following Shimadzu modules (Shimadzu Corporation, Marne-la-Vallée, France): a binary pump consisting of coupling two isocratic pumps Nexera LC30AD, an automated sampler SIL-30AC, a column oven CTO-20AC, and a triple-quadrupole mass spectrometer LCMS-8060 operating in the negative ion mode. Chromatographic separation was achieved on a Kinetex C18 column (2.6 μm particle size, 50 mm length × 3 mm inner diameter; Phenomenex, Le Pecq, France) maintained at 50 °C and gradient-elution chromatography using the following mobile phases: water with 0.01% acetic acid (A) and MeOH (B) at a flow rate of 0.600 mL/min. Gradient was as follows: 0.0–0.5 min, 10% (B); 0.5–2.0 min, 10 to 70% (B); 2.0–5.0 min, 70 to 75% (B); 5.0–5.1 min, 75 to 98% (B); 5.1–6.9 min, 98% (B); 6.9–7.0 min, 98% to 10% (B); and 7.0–8.0 min, 10% (B). The source interface parameters and common settings were as follows: interface voltage: −3 kV; nebulizing gas flow: 3 L/min; heating gas flow: 10 L/min; drying gas flow: 10 L/min; interface temperature: 400 °C; DL (desolvation line) temperature: 250 °C; heat block temperature: 500 °C; and collision gas pressure: 300 kPa. Detection and quantification were performed by scheduled- MRM using a pause time of 1 ms and individual dwell times to achieve sufficient points per peak. Mass transitions (*m*/*z*) and mass spectrometer parameters are summarized in Table S1.

## Supplementary Material



## Notes

Synthetic preparation, purification, and analytical characterization of all test compounds and their intermediates; DNA and protein sequence of msEH-P-p7XNH3; mass spectrometry parameters and summary of noncompartmental analysis parameters for analysis of the in vivo data; and HPLC chromatogram and NMR spectra of compound **49** (PDF)

## Abbreviations

Ac: acetyl
ACN: acetonitrile
AUC: area under the curve
aq: aqueous
BINAP: 2,2′-bis­(diphenylphosphino)-1,1′-binaphthyl
CETSA: Cellular thermal shift assay
Cas: CRISPR associated protein 9
CL_int_: intrinsic clearance
COX: cyclooxygenase
CRISPR: clustered regularly interspaced short palindromic repeats
dba: dibenzylideneacetone
DiFMUP: 6,8-difluoro-4-methylumbelliferyl phosphate
DIPEA: diisopropylethylamine
DMF: dimethylformamide
DMSO: dimethyl sulfoxide
DPBS: Dulbeccós phosphate-buffered saline
DSF: differential scanning fluorimetry
EDTA: ethylenediaminetetraacetic acid
EET: epoxyeicosatrienoic acid
EpDPA: epoxydocosapentaneoic acid
EpETE: epoxyeicosatetraenoic acid
Et: ethyl
ESI: electrospray ionization
FDP: fluorogenic fluorescein diphosphate
HAC: heavy atom count
HMDS: hexamethyldisilazane
HPLC: high-performance liquid chromatography
HRMS: high-resolution mass spectrometry
IC_50_: half maximal inhibitory concentration
IS: internal standard
LC: liquid chromatography
LE: ligand efficiency
LPP: lipid phosphate phosphatase
LPA: lysophosphatidic acid
MALDI: matrix-assisted laser desorption/ionization
Me: methyl
MRM: multiple reaction monitoring
MS: mass spectrometry
MW: microwave
NADP: nicotinamide adenine dinucleotide phosphate
NMR: nuclear magnetic resonance
p.o.: peroral
PCR: polymerase chain reaction
PHOME: cyano­(6-methoxy-2-naphthalenyl)­methyl 3-phenyloxiran-2-ylacetate
s.c.: subcutaneous
sEH: soluble epoxide hydrolase
sEH-FL: full-length protein of sEH
sEH-FL (H): hydrolase activity of sEH-FL
sEH-FL (P): phosphatase activity of sEH-FL
sEH-H: sEH hydrolase activity
sEH-P: sEH phosphatase activity
sEH-PD: sEH phosphatase domain
SEM: standard error of mean
sn1-16:0 LPA: 1-palmitoyl LPA
sn1-17:1 LPA: 1-heptadecenoyl LPA
sn1-18:0 LPA: 1-steraoyl LPA
sn1-18:1 LPA: 1-oleyl LPA
SPE: solid phase extraction
TBACl: tetrabutylammonium chloride
THF: tetrahydrofuran
TLC: thin layer chromatography
*T*_m_: melting temperature
UPLC: ultraperformance liquid chromatography
UV: ultraviolet
