# Supplementary material for: Fragment-Based Discovery of Fenamic Acids as Pan-Species Inhibitors of the Soluble Epoxide Hydrolase Phosphatase Domain
Source: ACS Chem Biol. 2026 Jul 28;21(8):2053–67. doi: 10.1021/acschembio.6c00502 (PMC13505187; doi:10.1021/acschembio.6c00502)
Supplement: Supplementary file 1 [file cb6c00502_si_001.pdf]

Supporting Information

Fragment-Based Discovery of Fenamic Acids as Pan-Species Inhibitors of the Soluble Epoxide Hydrolase Phosphatase Domain

W. Felix Zhu[1], Guillaume Feugray[2,3], Steffen Brunst[1], Hind Messaoudi[2], Meng Yang[4], Noemi Martinez-Conde[1,5], Andreas Krämer[1,6], Lilia Weizel[1], Johanna M. H. Ehrler[1], Jan S. Kramer[1], Astrid Kaiser[1], Mohammad Al-Tarrass[2], Tony Pereira[7], Manon Valet[2], Valéry Brunel[3], Manfred Schubert-Zsilavecz[1], Stefan Knapp[1,6], Felix F. Lillich,[1] Victor Hernandez-Olmos,[9] Thomas Duflot[2,7,8], Kerstin Hiesinger[1], Hongwu Qian[4], Anna Proschak[1], Jeremy Bellien[2,3,8], , Ewgenij Proschak[1,9,*]

[1] Institute of Pharmaceutical Chemistry, Goethe University, 60438 Frankfurt am Main, Germany

[2] Univ Rouen Normandie, Inserm U1096 EnVI, F-76000 Rouen, France

[3] Department of Biochemistry, CHU Rouen, F-76000 Rouen, France

[4]Department of Cardiology, The First Affiliated Hospital of USTC, MOE Key Laboratory for Membraneless Organelles and Cellular Dynamics, Hefei National Research Center for Interdisciplinary Sciences at the Microscale, Division of Life Sciences and Medicine, University of Science and Technology of China, Hefei 230027, China

[5] Laboratory of Medicinal Chemistry (CSIC Associated Unit), Faculty of Pharmacy and Food Sciences, and Institute of Biomedicine (IBUB), University of Barcelona (UB), E-08028 Barcelona, Spain

[6] Structural Genomics Consortium (SGC), Buchmann Institute for Molecular Life Sciences (BMLS), 60438 Frankfurt am Main, Germany

[7] Department of Pharmacology, CHU Rouen, F-76000 Rouen, France

[8] CIC-CRB 1404, CHU Rouen, F-76000 Rouen, France

[9] Fraunhofer Institute for Translational Medicine and Pharmacology ITMP, 60596, Frankfurt am Main, Germany

*proschak@pharmchem.uni-frankfurt.de

Table of content

Supplementary Methods 3

Chemistry 3

DNA and protein sequence of msEH-P-p7XNH3 13

Supplementary Tables 14

Supplementary Table 1. Mass spectrometry parameters for FZ581, LPAs and epoxyfatty acids. 14

Supplementary Table 2. Summary of non-compartmental analysis (NCA) parameters. 14

Supplementary Figures 15

Supplementary Figure 1. Stability of compound **49** and **46** in rat liver microsomes (n = 3). 15

Supplementary Figure 2. Evolution of plasma levels of FZ581 in C57BL/6 mice (n = 4) during 6 hours after intravenous administration at 1 mg/kg. 16

Supplementary Figure 3. Plasma levels of 1-palmitoyl LPA (sn1-16:0 LPA), 1-steraoyl LPA (sn1-18:0 LPA) and 1-oleyl LPA (sn1-18:1 LPA) at baseline and 6 hours after administration of FZ581 (60) at 1 mg/kg (square) and 10 mg/kg (triangle) in rats. 16

Supplementary Figure 4. HPLC Chromatogram of FZ581. 17

Supplementary Figure 5. NMR spectra of FZ581 18

Supplementary Methods

Chemistry

General Information

Reactions were carried out under argon atmosphere. NMR spectra were recorded with Bruker Avance DPX250, Bruker Avance 300, Bruker Avance 400, or Bruker Avance 500 all from Bruker (Karlsruhe, Germany) operating at ambient temperature. Proton spectra were recorded in acetone-d_6, $CDCl_3$ or DMSO-d_6 and ^1H NMR chemical shifts were referenced to the residual signals of acetone-d_5 (at δ = 2.05 ppm), $CHCl_3$ (at δ = 7.26 ppm) and DMSO-d_5 (at δ = 2.50 ppm). ^{13}C NMR chemical shifts were referenced against the central line of the solvent signal (for acetone-d_6 at δ = 29.84 ppm, for $CDCl_3$ at δ = 77.16 ppm and for DMSO-d_6 at δ = 39.52 ppm). Chemical shifts are given on δ scale (ppm). Coupling constants (J) are given in Hz. Multiplicities are indicated as follows: s (singlet), d (doublet), t (triplet), q (quartet), quint or m (multiplet). ESI-MS were measured with LCMS-2020 from Shimadzu and HRMS with MALDI Orbitrap XL from Thermo Scientific. TLC was carried out on silica gel plates from Marcherey-Nagel (ALUGRAM®) and visualized with an UV lamp (254 nm and/or 366 nm). Purification of products by flash chromatography were performed by using puriFlash XS420 and Silica HP 30 µm columns as stationary phase all from Interchim (Montluçon, France). Analytical and semipreparative HPLC was conducted by Shimadzu prominence with a SPD20A UV/Vis detector both from Shimadzu (Duisburg, Germany). Stationary phases were Luna 10 µm 100 Å, C18(2) (250 x 4.6 mm) and Luna 10 µm 100 Å, C18(2) (250 x 21.20 mm) from Phenomenex (Aschaffenburg, Germany) and eluent was a mixture of ACN and aq formic acid solution (0.1%). Flow rate was set to 1 mL/min and 21 mL/min. All compounds tested display a purity of >95% (254 nm).

General procedure 1 (GP1): Synthesis of fenamic acid derivatives via Ullmann coupling

The corresponding 2-bromobenzoic acid (1.0 equiv.), the corresponding aniline (2.0 equiv.), K_3PO_4 (1.0 equiv.), $Cu(OAc)_2$ (0.14 equiv.) and EtOH (0.8 M referred to 2-bromobenzoic acid derivative) were transferred to a microwave vessel, that was sealed afterwards and then irradiated with microwaves at 110 °C for 0.5 h (unless otherwise stated). Once the reaction mixture cooled down to rt, it was diluted with H_2O. The addition of an aq HCl solution (2 M) led to the precipitation of the crude product. The precipitate was purified by column or flash chromatography (*n*-hexane/EtOAc), unless otherwise stated.

General procedure 2 (GP2): Synthesis of fenamic acid derivatives via S_NAr

The corresponding 2-fluorobenzoic acid (1.0 equiv.) and the corresponding aniline (1.1-2.0 equiv.) were dissolved in THF (0.1 M). A LiHMDS solution in THF (1.3 M, 3 equiv) was added dropwise at -78 °C. Once the reaction mixture warmed up to rt, it was heated to reflux in an oil bath for 2 h. After that time, the reaction mixture was diluted with an aq HCl solution (2 M) and EtOAc. The organic phase was washed 1 x with an aq HCl solution, 1 x with an aq saturated NaCl solution, dried over MgSO4, and concentrated under reduced pressure. The crude product was purified by flash chromatography (DCM/MeOH), followed by preparative HPLC. Of note, the quality of the LiHMDS batch was crucial for reaction outcome.

2-[(3-Chlorophenyl)amino]benzoic acid (5)

Synthesized according to GP1 from 2-bromobenzoic acid (375 mg, 1.86 mmol) and 3-chloroaniline (400 µL, 3.73 mmol). The addition of an aq HCl solution (2 M) led to the precipitation of the product as a yellow solid (406 mg, 84%). ^1H NMR (500 MHz, DMSO-d_6) δ 9.61 (s, 1H), 7.92 (dd, J = 8.0, 1.5 Hz,

1H), 7.45 (td, J = 7.1, 1.7 Hz, 1H), 7.37-7.29 (m, 1H), 7.28 (d, J = 1.9 Hz, 1H), 7.20 (ddd, J = 8.1, 2.1, 1.0 Hz, 1H), 7.06 (ddd, J = 7.9, 2.0, 1.0 Hz, 1H), 6.86 (td, J = 7.1, 1.1 Hz, 1H).

2-[(3-Chlorophenyl)amino]-5-methylbenzoic acid (6)

2-Bromo-5-methylbenzoic acid (150 mg, 0.677 mmol), 3-chloroaniline (1.40 mL, 0.784 mmol), K_2CO_3 (1.31 g, 9.47 mmol), and Cu (24 mg, 0.372 mmol) were suspended in EtOH (6.0 mL) and heated in an oil bath to reflux for 7 h. After that time, the reaction mixture was filtered over celite. The addition of an aq HCl solution (2 M) led to the precipitation of the crude product. Purification of the crude product by column chromatography (*n*-hexane/EtOAc) yielded the title compound as a yellow solid (45 mg, 25%). ^{1}H NMR (500 MHz, DMSO-d_6) δ 13.08 (s, 1H), 9.43 (s, 1H), 7.73 (s, 1H), 7.32-7.22 (m, 4H), 7.15-7.13 (m, 1H), 7.00-6.99 (m, 1H), 2.24 (s, 3H). ^{13}C{^{1}H} NMR (126 MHz, DMSO-d_6) δ 170.1, 143.7, 143.5, 135.3, 134.2, 132.2, 131.4, 128.2, 121.9, 119.1, 118.3, 116.2, 115.1, 20.5. MALDI-HRMS: *m/z* calculated for $C_{14}H_{12}ClNO_2$[radical]: 261.0551, found: 261.0551.

2-[(3-Chlorophenyl)amino]-4-methylbenzoic acid (7)

Synthesized according to GP1 from 2-bromo-4-methylbenzoic acid (85 mg, 0.392 mmol) and 3-chloroaniline (83 µL, 0.784 mmol) and a reaction temperature of 90 °C. Purification of the crude product by column chromatography (*n*-hexane/EtOAc) yielded the title compound as a yellow solid (40 mg, 39%). ^{1}H NMR (500 MHz, DMSO-d_6) δ 12.99 (s, 1H), 9.64 (s, 1H), 7.81 (d, J = 8.1 Hz, 1H), 7.34 (t, J = 8.1 Hz, 1H), 7.27 (t, J = 2.0 Hz, 1H), 7.22-7.20 (m, 1H), 7.10 (s, 1H), 7.07-7.05 (m, 1H), 6.68 (dd, J = 8.2, 0.8 Hz, 1H), 2.26 (s, 3H). ^{13}C{^{1}H} NMR (126 MHz, DMSO-d_6) δ 169.7, 145.83, 144.60, 142.64, 133.8, 131.91, 130.99, 122.15, 120.1, 119.8, 118.9, 114.8, 111.3, 21.5. MALDI-HRMS: *m/z* calculated for $C_{14}H_{12}ClNO_2$[radical]: 261.0554, found: 261.0551.

2-[(3-Chlorophenyl)amino]-5-methoxybenzoic acid (8)

Synthesized according to GP1 from 2-bromo-5-methoxybenzoic acid (92 mg, 0.392 mmol) and 3-chloroaniline (83 µL, 0.784 mmol). Purification of the crude product by column chromatography (*n*-hexane/EtOAc) yielded the title compound as a yellow solid (31 mg, 28%). ^{1}H NMR (500 MHz, DMSO-d_6) δ 13.25 (s, 1H), 10.10 (s, 1H), 7.62 (d, J = 3.1 Hz, 1H), 7.31 (d, J = 9.1 Hz, 1H), 7.26 (t, J = 8.1 Hz, 1H), 7.14-7.12 (m, 2H) 7.06 (dd, J = 8.2, 1.4 Hz, 1H), 6.92 (dd, J = 7.9, 1.1 Hz, 1H), 3.75 (s, 3H). ^{13}C{^{1}H} NMR (126 MHz, DMSO-d_6) δ 169.0, 152.3, 144.4, 138.4, 133.8, 130.9, 121.1, 120.4, 119.1, 117.4, 117.1, 116.5, 114.8, 55.4. MALDI-HRMS: *m/z* calculated for $C_{14}H_{12}ClNO_2$[radical]: 277.0501, found: 277.0500.

2-[(3-Chlorophenyl)amino]-4-methoxybenzoic acid (9)

Synthesized according to GP1 from 2-bromo-4-methoxybenzoic acid (92 mg, 0.392 mmol) and 3-chloroaniline (83 µL, 0.784 mmol). Purification of the crude product by column chromatography (*n*-hexane/EtOAc) yielded the title compound as a colorless solid (49 mg, 46%). ^{1}H NMR (500 MHz, DMSO-d_6) δ 12.84 (s, 1H), 9.82 (s, 1H), 7.87 (d, J = 8.9 Hz, 1H), 7.36 (t, J = 8.0 Hz, 1H), 7.32 (t, J = 2.0 Hz, 1H), 7.27-7.25 (m, 1H), 7.09 (dd, J = 8.2, 1.9 Hz, 1H), 6.71 (d, J = 2.4 Hz, 1H), 6.45 (dd, J = 8.9, 2.4 Hz, 1H), 3.74 (s, 3H). ^{13}C{^{1}H} NMR (126 MHz, DMSO-d_6) δ 169.5, 163.8, 147.8, 142.3, 133.9, 133.9, 131.1, 122.5, 120.5, 119.3, 106.5, 105.3. 98.5, 55.3. MALDI-HRMS: *m/z* calculated for $C_{14}H_{12}ClNO_2$[radical]: 277.0501, found: 277.0500.

2-[(3-Chlorophenyl)amino]-5-fluorobenzoic acid (10)

Synthesized according to GP1 from 2-bromo-5-fluorobenzoic acid (88 mg, 0.392 mmol) and 3-chloroaniline (83 µL, 0.784 mmol). Purification of the crude product by column chromatography (*n*-hexane/EtOAc) yielded the title compound as a yellow solid (51 mg, 49%). ^{1}H NMR (500 MHz,

DMSO-d_6) δ 9.41 (s, 1H), 7.62 (dd, J = 9.7, 2.8 Hz, 1H), 7.35-7.30 (m, 3H), 7.24 (t, J = 2.1 Hz, 1H), 7.15 (dd, J = 8.1, 2.0 Hz, 1H), 7.03 (dd, J = 8.0, 1.3 Hz, 1H). $^{13}C\{^1H\}$ NMR (126 MHz, DMSO-d_6) δ 168.5, 154.6, 143.2, 142.0, 133.8, 131.0, 121.8, 121.4, 119.1, 118.1, 117.7, 117.1, 116.0. MALDI-HRMS: *m/z* calculated for $C_{13}H_9ClFNO_2$[radical]: 265.0300, found: 265.0300.

2-[(3-Chlorophenyl)amino]-4-fluorobenzoic acid (11)

Synthesized according to GP1 from 2-bromo-4-fluorobenzoic acid (90 mg, 0.392 mmol) and 3-chloroaniline (83 µL, 0.784 mmol). Purification of the crude product by column chromatography (*n*-hexane/EtOAc) yielded the title compound as a colorless solid (58 mg, 56%). ^1H NMR (500 MHz, DMSO-d_6) δ 9.87 (s, 1H), 7.99 (dd, J = 7.0, 1.9 Hz, 1H), 7.39 (t, J = 8.1 Hz, 1H), 7.35 (t, J = 2.0 Hz, 1H), 7.30-7.26 (m, 1H), 7.19-7.13 (m, 1H), 6.93 (dd, J = 8.1, 1.3 Hz, 1H), 6.66 (td, J = 8.7, 2.5 Hz, 1H). $^{13}C\{^1H\}$ NMR (126 MHz, DMSO-d_6) δ 169.5, 166.2, 148.9, 142.0, 135.3, 134.4, 131.6, 123.9, 121.7, 120.5, 110.4, 106.0, 100.6. MALDI-HRMS: *m/z* calculated for $C_{13}H_9ClFNO_2$[radical]: 265.0300, found: 265.0300.

5-Chloro-2-[(3-chlorophenyl)amino]benzoic acid (12)

Synthesized according to GP1 from 2-bromo-5-chlorobenzoic acid (94 mg, 0.392 mmol) and 3-chloroaniline (83 µL, 0.784 mmol). Purification of the crude product by column chromatography (*n*-hexane/EtOAc) yielded the title compound as a yellow solid (40 mg, 36%). ^1H NMR (500 MHz, DMSO-d_6) δ 9.58 (s, 1H), 7.84 (d, J = 2.7 Hz, 1H), 7.46 (dd, J = 9.0, 2.7 Hz, 1H), 7.35 (t, J = 8.1 Hz, 1H), 7.30 (t, J = 2.0 Hz, 1H), 7.28 (d, J = 9.0 Hz, 1H), 7.21-7.19 (m, 1H), 7.11-7.09 (m, 1H). $^{13}C\{^1H\}$ NMR (126 MHz, DMSO-d_6) δ 168.5, 144.7, 142.2, 133.8, 133.8, 131.0, 130.8, 122.7, 121.6, 120.4, 119.3, 116.8, 115.3. MALDI-HRMS: *m/z* calculated for $C_{13}H_9Cl_2NO_2$[radical]: 281.0005, found: 281.0006.

4-Chloro-2-[(3-chlorophenyl)amino]benzoic acid (13)

Synthesized according to GP1 from 2-bromo-4-chlorobenzoic acid (95 mg, 0.392 mmol) and 3-chloroaniline (83 µL, 0.784 mmol). Purification of the crude product by column chromatography (*n*-hexane/EtOAc) yielded the title compound as a yellow solid (21 mg, 19%). ^1H NMR (500 MHz, DMSO-d_6) δ 13.39 (s, 1H), 9.74 (s, 1H), 7.91 (d, J = 8.5 Hz, 1H), 7.40 (t, J = 8.1 Hz, 1H), 7.35 (t, J = 2.0 Hz, 1H), 7.28-7.26 (m, 1H), 7.16 (dd, J = 8.0, 1.3 Hz, 1H), 7.14 (d, J = 2.0 Hz, 1H), 6.87 (dd, J = 8.6, 2.0 Hz, 1H). $^{13}C\{^1H\}$ NMR (126 MHz, DMSO-d_6) δ 169.1, 147.3, 141.6, 138.8, 133.9, 133.7, 131.2, 123.5, 121.5, 120.2, 118.1, 113.3, 112.3. MALDI-HRMS: *m/z* calculated for $C_{13}H_9Cl_2NO_2$[radical]: 281.0005, found: 281.0006.

2-[(3-Chlorophenyl)amino]-5-cyanobenzoic acid (14)

Synthesized according to GP1 from 2-bromo-5-cyanobenzoic acid (91 mg, 0.392 mmol) and 3-chloroaniline (83 µL, 0.784 mmol). Purification of the crude product by preparative HPLC yielded the title compound as a yellow solid (5 mg, 5%). ^1H NMR (500 MHz, DMSO-d_6) δ 10.31 (s, 1H), 8.24 (d, J = 2.0 Hz, 1H), 7.74 (dd, J = 8.9, 2.0 Hz, 1H), 7.41 (t, J = 2.0 Hz, 1H), 7.30 (t, J = 8.1 Hz, 1H), 7.30 (dd, J = 8.1, 1.3 Hz, 1H), 7.26-7.24 (m, 1H), 7.23 (d, J = 8.9 Hz, 1H). $^{13}C\{^1H\}$ NMR (126 MHz, DMSO-d_6) δ 168.4, 149.7, 140.6, 136.9, 136.6, 133.9, 131.2, 124.6, 122.7, 121.6, 119.0, 114.3, 113.7, 99.0. MALDI-HRMS: *m/z* calculated for $C_{14}H_9ClN_2O_2$[radical]: 272.0351, found: 272.0347.

2-[(3-Chlorophenyl)amino]-4-cyanobenzoic acid (15)

Synthesized according to GP1 from 2-bromo-4-cyanobenzoic acid (91 mg, 0.392 mmol) and 3-chloroaniline (83 µL, 0.784 mmol). Purification of the crude product by preparative HPLC yielded the title compound as a yellow solid (26 mg, 24%). ^1H NMR (500 MHz, DMSO-d_6) δ 9.74 (s, 1H), 8.03 (d, J = 8.1 Hz, 1H), 7.53 (s, 1H), 7.40 (t, J = 7.4 Hz, 1H), 7.36 (d, J = 1.9 Hz, 1H), 7.28 (d, J = 8.0 Hz, 1H),

7.21 (d, J = 8.1 Hz, 1H), 7.17 (d, J = 7.9 Hz, 1H). $^{13}C\{^1H\}$ NMR (126 MHz, DMSO-d_6) δ 168.6, 146.1, 141.4, 133.9, 132.8, 131.1, 123.5, 121.3, 120.7, 119.9, 118.1, 117.7, 117.4, 116.0. MALDI-HRMS: *m/z* calculated for $C_{14}H_9ClN_2O_2$[radical]: 272.0351, found: 272.0347.

2-[(3-Chlorophenyl)amino]-5-(trifluoromethyl)benzoic acid (16)

Synthesized according to GP1 from 2-bromo-5-(trifluoromethyl)benzoic acid (105 mg, 0.392 mmol) and 3-chloroaniline (83 µL, 0.784 mmol) and a reaction temperature of 90 °C. Purification of the crude product by column chromatography (*n*-hexane/EtOAc) yielded the title compound as a brown solid (46 mg, 38%). ^1H NMR (500 MHz, DMSO-d_6) δ 10.10 (s, 1H), 8.15 (d, J = 1.9 Hz, 1H), 7.71-7.67 (m, 1H), 7.43-7.39 (m, 2H), 7.32 (d, J = 8.9 Hz, 1H), 7.29 (dd, J = 7.5, 1.3 Hz, 1H), 7.20 (dd, J = 7.9, 1.8 Hz, 1H). $^{13}C\{^1H\}$ NMR (126 MHz, DMSO-d_6) δ 168.8, 149.2, 141.1, 133.9, 131.2, 129.7, 127.6, 124.4, 124.0, 122.1, 121.0, 117.2, 114.4, 113.0. MALDI-HRMS: *m/z* calculated for $C_{14}H_9ClF_3NO_2$[radical]: 315.0268, found: 315.0268.

2-[(3-Chlorophenyl)amino]-4-(trifluoromethyl)benzoic acid (17)

Synthesized according to GP1 from 2-bromo-4-(trifluoromethyl)benzoic acid (105 mg, 0.392 mmol) and 3-chloroaniline (83 µL, 0.784 mmol). Purification of the crude product by column chromatography (*n*-hexane/EtOAc) yielded the title compound as a yellow solid (13 mg, 11%). ^1H NMR (500 MHz, DMSO-d_6) δ 13.68 (s, 1H), 9.73 (s, 1H), 8.09 (d, J = 8.2 Hz, 1H), 7.40 (t, J = 8.1 Hz, 1H), 7.38-7.38 (m, 1H), 7.36 (t, J = 2.1 Hz, 1H), 7.28-7.26 (m, 1H), 7.18-7.16 (m, 1H), 7.13 (dd, J = 8.3, 1.2 Hz, 1H). $^{13}C\{^1H\}$ NMR (126 MHz, DMSO-d_6) δ 168.8, 146.4, 141.6, 134.0, 133.6, 133.3, 131.2, 124.5, 123.6, 121.5, 120.1, 116.8, 114.1, 110.5. MALDI-HRMS: *m/z* calculated for $C_{14}H_9ClF_3NO_2$[radical]: 315.0267, found: 315.0268.

4-Chloro-2-[(3-chlorophenyl)amino]-5-sulfamoylbenzoic acid (18)

Synthesized according to GP2 from 4-chloro-2-fluoro-5-sulfamoylbenzoic acid (100 mg, 0.386 mmol) and 3-chloroaniline (83 µL, 0.773 mmol). Purification of the crude product by flash chromatography (DCM/MeOH), followed by preparative HPLC yielded the title compound as a purple solid (50 mg, 36%). ^1H NMR (500 MHz, DMSO-d_6) δ 10.00 (s, 1H), 8.49 (s, 1H), 7.48-7.42 (m, 4H), 7.33 (dd, J = 8.0, 1.2 Hz, 1H), 7.26 (dd, J = 8.0, 1.2 Hz, 1H), 7.14 ppm (s, 1H). $^{13}C\{^1H\}$ NMR (126 MHz, DMSO-d_6) δ 168.5, 149.5, 140.4, 136.1, 134.0, 133.3, 131.3, 129.6, 125.0, 123.1, 121.8, 115.2, 110.5. MALDI-HRMS: *m/z* calculated for $C_{13}H_{10}Cl_2N_2O_4S$[radical]: 359.9733, found: 359.9733.

2-[(6-Chloropyridin-2-yl)amino]benzoic acid (19)

Synthesized according to GP1 from 2-bromobenzoic acid (79 mg, 0.392 mmol) and 6-chloropyridin-2-amine (103 mg, 0.784 mmol) and a reaction temperature of 120 °C. Purification of the crude product by column chromatography (*n*-hexane/EtOAc) yielded the title compound as a brown solid (18 mg, 19%). ^1H NMR (500 MHz, DMSO-d_6) δ 13.42 (s, 1H), 10.76 (s, 1H), 8.45 (d, J = 8.5 Hz, 1H), 7.98 (dd, J = 7.9, 1.3 Hz, 1H), 7.68 (t, J = 7.9 Hz, 1H), 7.60-7.57 (m, 1H), 7.01 (t, J = 7.5 Hz, 1H), 6.96-6.95 (m, 2H). $^{13}C\{^1H\}$ NMR (126 MHz, DMSO-d_6) δ 170.0, 154.3, 147.8, 142.9, 140.8, 134.1, 131.4, 120.3, 118.2, 115.1, 115.0, 110.6. MALDI-HRMS: *m/z* calculated for $C_{12}H_{10}Cl_2N_2O_2[M+H]^+$: 249.0429, found: 249.0425.

2-(Quinolin-2-ylamino)benzoic acid (20)

2-Aminobenzoic acid (137 mg, 1.00 mmol), 2-chloroquinoline (150 mg, 0.908 mmol) and conc HCl (0.10 mL) were suspended in EtOH (5.0 mL) and heated in an oil bath to 50 °C overnight. Dilution of the reaction mixture with H_2O, followed by the addition of an aq HCl solution (2 M) led to the precipitation of the crude product. Purification of the crude product by column chromatography

(toluene/EtOH) yielded the title compound as a yellow oil (60 mg, 25%). ^{1}H NMR (500 MHz, DMSO-d_6) δ 11.15 (s, 1H), 9.27 (d, J = 8.2 Hz, 1H), 8.16 (d, J = 8.8 Hz, 1H), 8.02 (dd, J = 7.9, 1.5 Hz, 1H), 7.82-7.78 (m, 2H), 7.65-7.62 (m, 2H), 7.38-7.35 (m, 1H), 7.10 (d, J = 8.8 Hz, 1H), 7.05-7.01 (m, 1H). ^{13}C{^{1}H} NMR (126 MHz, DMSO-d_6) δ 170.3, 153.1, 146.6, 143.7, 137.7, 134.1, 131.3, 129.8, 127.6, 126.7, 124.0, 123.6, 120.0, 118.8, 115.0, 114.7. MALDI-HRMS: *m/z* calculated for $C_{16}H_{13}N_2O_2$[M+H]$^+$: 265.0977, found: 265.0972.

2-[(1*H*-Indol-6-yl)amino]benzoic acid (21)

Synthesized according to GP1 from 2-bromobenzoic acid (79 mg, 0.392 mmol) and 6-aminoindole (107 mg, 0.784 mmol). Purification of the crude product by crystallization (*n*-hexane+EtOAc) yielded the title compound as a dark brown solid (38 mg, 39%). ^{1}H NMR (500 MHz, DMSO-d_6) δ 13.91 (s, 1H), 11.01 (s, 1H), 9.60 (s, 1H), 7.88 (d, J = 7.3 Hz, 1H), 7.53 (d, J = 8.3 Hz, 1H), 7.34-7.28 (m, 3H), 7.09 (d, J = 8.5 Hz, 1H), 6.88 (dd, J = 8.4, 1.8 Hz, 1H), 6.68 (t, J = 7.4 Hz, 1H), 6.41-6.41 (m, 1H). ^{13}C{^{1}H} NMR (126 MHz, DMSO-d_6) δ 170.1, 149.0, 136.5, 134.2, 133.9, 131.8, 125.4, 125,2, 124.9, 120.8, 116.2, 113.1, 111.2, 105.8, 101.1. MALDI-HRMS: *m/z* calculated for $C_{15}H_{12}N_2O_2$[radical]: 252.2895, found: 252.2893.

2-[(2-Fluorophenyl)amino]benzoic acid (22)

Synthesized according to GP1 from 2-bromobenzoic acid (79 mg, 0.392 mmol) and 2-fluoroaniline (76 µL, 0.784 mmol). Purification of the crude product by column chromatography (*n*-hexane/EtOAc) yielded the title compound as a yellow solid (61 mg, 67%). ^{1}H NMR (500 MHz, DMSO-d_6) δ 13.19 (s, 1H), 9.65 (s, 1H), 7.92 (dd, J = 8.0, 1.6 Hz, 1H), 7.51 (td, J = 8.3, 1.7 Hz, 1H), 7.42 (td, J = 7.2, 1.7 Hz, 1H), 7.31 (td, J = 8.1, 1.5 Hz, 1H), 7.20 (td, J = 7.7, 1.4 Hz, 1H), 7.15-7-12 (m, 1H), 7.31 (d, J = 8.5 Hz, 1H), 6.84-6.81 (m, 1H). ^{13}C{^{1}H} NMR (126 MHz, DMSO-d_6) δ 170.0, 154.9, 146.4, 134.3, 131.8, 128.3, 125.0, 124.4, 123.1, 118.0, 116.2, 113.8, 112.9. MALDI-HRMS: *m/z* calculated for $C_{13}H_{10}FNO_2$[radical]: 231.0694, found: 231.0690.

2-[(3-Fluorophenyl)amino]benzoic acid (23)

Synthesized according to GP1 from 2-bromobenzoic acid (79 mg, 0.392 mmol) and 3-fluoroaniline (76 µL, 0.784 mmol). Purification of the crude product by column chromatography (*n*-hexane/EtOAc) yielded the title compound as a yellow solid (18 mg, 20%). ^{1}H NMR (500 MHz, DMSO-d_6) δ 13.18 (s, 1H), 9.67 (s, 1H), 7.92 (dd, J = 8.0, 1.6 Hz, 1H), 7.44 (td, J = 7.1, 1.7 Hz, 1H), 7.37-7.32 (m, 2H), 7.10-7.05 (m, 2H), 6.88-6.81 (m, 2H). ^{13}C{^{1}H} NMR (126 MHz, DMSO-d_6) δ 169.8, 162.9, 145.7, 142.9, 134.2, 131.9, 131.0, 118.6, 116.2, 114.9, 113.9, 108.9, 106.9. MALDI-HRMS: *m/z* calculated for $C_{13}H_{10}FNO_2$[radical]: 231.0694, found: 231.0690.

2-[(4-Fluorophenyl)amino]benzoic acid (24)

Synthesized according to GP1 from 2-bromobenzoic acid (79 mg, 0.392 mmol) and 4-fluoroaniline (75 µL, 0.784 mmol). Purification of the crude product by column chromatography (*n*-hexane/EtOAc) yielded the title compound as a yellow solid (78 mg, 88%). ^{1}H NMR (500 MHz, DMSO-d_6) δ 13.01 (s, 1H), 9.54 (s, 1H), 7.89 (dd, J = 8.0, 1.6 Hz, 1H), 7.36 (td, J = 7.1, 1.7 Hz, 1H), 7.29-7.26 (m, 2H), 7.21-7.17 (m, 2H), 7.06 (dd, J = 8.5, 0.7 Hz, 1H), 6.77-6.74 (m, 1H). ^{13}C{^{1}H} NMR (126 MHz, DMSO-d_6) δ 170.0, 158.2, 147.7, 136.8, 134.3, 131.9, 124.3, 117.2, 116.2, 113.3, 112.2. MALDI-HRMS: *m/z* calculated for $C_{13}H_{10}FNO_2$[radical]: 231.0694, found: 231.0690.

2-[(2-Chlorophenyl]amino)benzoic acid (25)

Synthesized according to GP1 from 2-bromobenzoic acid (79 mg, 0.392 mmol) and 2-chloroaniline (84 µL, 0.784 mmol). Purification of the crude product by column chromatography (*n*-hexane/EtOAc)

yielded the title compound as a colorless solid (35 mg, 36%). ^{1}H NMR (500 MHz, DMSO-d$_6$) δ 13.23 (s, 1H), 9.83 (s, 1H), 7.94 (dd, J = 8.0, 1.7 Hz, 1H), 7.54 (dd, J = 8.1, 1.4 Hz, 1H), 7.54 (dd, J = 8.1, 1.5 Hz, 1H), 7.44 (td, J = 7.2, 1.7 Hz, 1H), 7.34-7.31 (m, 1H), 7.21 (d, J = 8.5 Hz, 1H), 7.08 (td, J = 7.9, 1.5 Hz, 1H), 6.89-6.85 (m, 1H). ^{13}C{^{1}H} NMR (126 MHz, DMSO-d$_6$) δ 168.8, 144.6, 136.5, 133.2, 130.9, 129.1, 127.0, 124.1, 122.9, 120.2, 117.5, 113.4, 112.7. MALDI-HRMS: *m/z* calculated for $C_{13}H_{10}ClNO_2$[radical]: 247.0401, found: 247.0395.

2-[(4-Chlorophenyl]amino)benzoic acid (26)

Synthesized according to GP1 from 2-bromobenzoic acid (79 mg, 0.392 mmol) and 4-chloroaniline (71 µL, 0.784 mmol). Purification of the crude product by column chromatography (*n*-hexane/EtOAc) yielded the title compound as an off-white solid (63 mg, 64%). ^{1}H NMR (500 MHz, DMSO-d$_6$) δ 13.14 (s, 1H), 9.62 (s, 1H), 7.91 (dd, J = 8.0, 1.6 Hz, 1H), 7.41 (td, J = 7.2, 1.7 Hz, 1H), 7.39-7.36 (m, 2H), 7.28-7.25 (m, 2H), 7.22 (d, J = 8.0 Hz, 1H), 6.82 (d, J = 7.7 Hz, 1H). ^{13}C{^{1}H} NMR (126 MHz, DMSO-d$_6$) δ 169.8, 146.3, 139.7, 134.2, 131.9, 129.3, 126.3, 122.6, 118.1, 114.2, 113.3. MALDI-HRMS: *m/z* calculated for $C_{13}H_{10}ClNO_2$[radical]: 247.0398, found: 247.0395.

2-(*p*-Tolylamino)benzoic acid (27)

Synthesized according to GP1 from 2-bromobenzoic acid (79 mg, 0.392 mmol) and 4-methylaniline (84 mg, 0.784 mmol). Purification of the crude product by column chromatography (*n*-hexane/EtOAc) yielded the title compound as brown solid (67 mg, 77%). ^{1}H NMR (500 MHz, DMSO-d$_6$) δ 13.02 (s, 1H), 9.56 (s, 1H), 7.88 (dd, J = 8.0, 1.6 Hz, 1H), 7.35 (td, J = 7.3, 1.7 Hz, 1H), 7.18-7.11 (m, 5H), 6.75-6.72 (m, 1H), 2.28 (s, 3H). ^{13}C{^{1}H} NMR (126 MHz, DMSO-d$_6$) δ 170.1, 147.7, 137.7, 134.2, 132.6, 131.9, 130.0, 122.2, 116.9, 113.3, 111.9, 20.5. MALDI-HRMS: *m/z* calculated for $C_{14}H_{14}NO_2$[radical]: 228.1015, found: 228.1019.

2-(Naphthalen-1-ylamino)benzoic acid (28)

Synthesized according to GP1 from 2-bromobenzoic acid (100 mg, 0.496 mmol) and 1-naphtylamine (144 mg, 0.993 mmol). Purification of the crude product by column chromatography (*n*-hexane/EtOAc) yielded the title compound as a brown solid (26 mg, 20%). ^{1}H NMR (500 MHz, DMSO-d$_6$) δ 10.08 (s, 1H), 8.01-7.95 (m, 3H), 7.77 (dd, J = 6.9, 2.1 Hz, 1H), 7.61-7.50 (m, 4H), 7.34-7.29 (m, 1H), 6.92 (d, J = 8.3 Hz, 1H), 6.77 (t, J = 7.5 Hz, 1H). ^{13}C{^{1}H} NMR (126 MHz, DMSO-d$_6$) δ 170.4, 148.6, 136.2, 134.4, 134.2, 131.8, 128.6, 128.5, 126.4, 126.1, 124.6, 121.8, 119.7, 117.1, 113.8, 112.2. MALDI-HRMS: *m/z* calculated for $C_{17}H_{13}NO_2$[radical]: 263.0941, found: 263.0944.

2-(Naphthalen-2-ylamino)benzoic acid (29)

Synthesized according to GP1 from 2-bromobenzoic acid (79 mg, 0.392 mmol) and 2-naphtylamine (112 mg, 0.784 mmol). Purification of the crude product by column chromatography (*n*-hexane/EtOAc) yielded the title compound as a brown solid (50 mg, 49%). ^{1}H NMR (500 MHz, DMSO-d$_6$) δ 13.17 (s, 1H), 9.85 (s, 1H), 7.95 (dd, J = 8.0, 1.5 Hz, 1H), 7.89 (d, J = 8.8 Hz, 1H), 7.85 (d, J = 8.1 Hz, 1H), 7.81 (d, J = 8.2 Hz, 1H), 7.73 (d, J = 1.7 Hz, 1H), 7.47-7.37 (m, 5H), 6.84 (t, J = 7.6 Hz, 1H). ^{13}C{^{1}H} NMR (126 MHz, DMSO-d$_6$) δ 170.0, 146.7, 138.3, 134.3, 134.0, 132.0, 129.7, 129.2, 127.6, 126.9, 126.5, 124.4, 122.2, 117.9, 115.9, 114.3, 113.1. MALDI-HRMS: *m/z* calculated for $C_{17}H_{13}NO_2$[radical]: 263.0947, found: 263.0941.

***N*-(3-Chlorophenyl)-2-(1*H*-tetrazol-5-yl)aniline (30)**

2-((3-Chlorophenyl)amino)benzonitrile (**66**) (100 mg, 0.438 mmol) and azidotributyltin(IV) (0.18 mL, 0.657 mmol) were suspended in *p*-xylene (5.0 mL) and heated in an oil bath to 150 °C overnight. After that time, the reaction mixture was diluted with EtOAc and the organic phase was washed 2 x

with an aq saturated NH_4Cl solution, 1 x with an aq saturated NaCl solution, dried over MgSO4, and concentrated under reduced pressure. Purification of the crude product by column chromatography (*n*-hexane/EtOAc/HOAc) yielded the title compound as a brown solid (23 mg, 19%). ^1H NMR (500 MHz, DMSO-d_6) δ 9.03 (s, 1H), 7.88 (dd, J = 7.9, 1.3 Hz, 1H), 7.50-7.47 (m, 1H), 7.45-7.43 (m, 1H) 7.29 (t, J = 8.1 Hz, 1H), 7.18 (t, J = 2.0 Hz, 1H), 7.12-7.09 (m, 2H), 6.98 (ddd, J = 7.9, 1.9, 0.7 Hz, 1H). $^{13}C\{^1H\}$ NMR (126 MHz, DMSO-d_6) δ 154.2, 143.8, 141.4, 133.8, 132.1, 130.9, 129.7, 121.1, 120.9, 118.1, 117.7, 117.2, 111.9. MALDI-HRMS: *m/z* calculated for $C_{13}H_{11}ClN_5[M+H]^+$: 272.0698, found: 272.0702.

2-[(3-Chlorophenyl)(methyl)amino]benzoic acid (31)

NaH was added portionwise to a solution of 2-((3-Chlorophenyl)amino)benzoic acid (**5**) (406 mg, 1.64 mmol) in DMF (5.0 mL) at 0 °C and then stirred at rt for 0.5 h. After that time, MeI (0.3 mL, 4.77 mmol) was added and the reaction mixture stirred at rt for 4 h. The reaction mixture was diluted with H2O and the aqueous phase was extracted 3 x with EtOAc. The organic phases were dried over MgSO4 and concentrated under reduced pressure. The residue was dissolved in MeOH (7.0 mL), an aq NaOH solution (2 M, 3.5 mL) was added and the resulting solution was heated to reflux in an oil bath for 3 h. The reaction mixture was concentrated under reduced pressure, dissolved with H_2O and extracted 3 x with EtOAc. The organic phases were dried over MgSO4 and concentrated under reduced pressure. Purification of the crude product by crystallization (*n*-hexane+EtOAc) yielded the title compound as a yellow solid (66 mg, 16%). ^1H NMR (500 MHz, DMSO-d_6) δ 12.81 (s, 1H), 7.83 (dd, J = 7.7, 1.6 Hz, 1H), 7.66 (dt, J = 7.6, 1.7 Hz, 1H), 7.42 (dt, J = 7.6, 1.1 Hz, 1H), 7.33 (dd, J = 8.1, 1.0 Hz, 1H), 7.10 (t, J = 8.1 Hz, 1H), 6.65 (dd, J = 7.7, 1.7 Hz, 2H), 6.46 (t, J = 2.3 Hz, 1H), 6.39 (dd, J = 8.6, 2.2 Hz, 1H), 3.19 (s, 3H). $^{13}C\{^1H\}$ NMR (126 MHz, DMSO-d_6) δ 167.4, 150.3, 146.0, 133.6, 133.5, 131.2, 130.8, 130.2, 129.9, 126.8, 116.2, 112.0, 111.4, 39.9. MALDI-HRMS: *m/z* calculated for $C_{14}H_{13}ClNO_2[M+H]^+$: 262.0630, found: 262.0629.

4-Chloro-2-(naphthalen-2-ylamino)-5-sulfamoylbenzoic acid (32)

Synthesized according to GP2 from 4-chloro-2-fluoro-5-sulfamoylbenzoic acid (100 mg, 0.386 mmol) and 2-naphtylamine (61 mg, 0.425 mmol). Purification of the crude product by preparative HPLC yielded the title compound as a colorless solid (15 mg, 10%). ^1H NMR (500 MHz, DMSO-d_6) δ 10.24 (s, 1H), 8.52 (s, 1H), 8.00 (d, J = 8.7 Hz, 1H), 7.95-7.87 (m, 3H), 7.54-7.45 (m, 5H), 7.21 (s, 1H). $^{13}C\{^1H\}$ NMR (126 MHz, DMSO-d_6) δ 169.1, 150.5, 136.7, 136.5, 134.2, 133.9, 131.2, 130.1, 129.5, 128.1, 127.8, 127.2, 126.0, 123.6, 120.5, 115.3, 110.5. MALDI-HRMS: *m/z* calculated for $C_{17}H_{12}ClNO_2$[radical]: 376.0279, found: 376.0281.

4-Chloronaphthalen-2-amine (52)

4-Bromonaphthalen-2-amine (117 mg, 0.500 mmol), tetrabutylammonium chloride (TBACl) (293 mg, 1.00 mmol), Cu_2O (7 mg, 0.050 mmol), and (*S*)-proline (12 mg, 0.100) and EtOH (1.0 mL) were transferred to a microwave vessel, that was sealed afterwards and heated in an oil bath to 130 °C for 15 days. After that time, the reaction mixture was filtered over celite. Purification of the crude product by column chromatography (*n*-hexane/EtOAc) yielded the title compound as a yellow oil (35 mg, 39%). ^1H NMR (400 MHz, $CDCl_3$) δ 8.12-8.09 (m, 1H), 7.61-7.58 (m, 1H), 7.44-7.32 (m, 2H), 7.08 (d, J = 2.2 Hz, 1H), 6.91-6.89 (m, 1H), 3.83-3.83 (s, 2H).

4-Chloro-2-[(4-chloronaphthalen-2-yl)amino]-5-sulfamoylbenzoic acid (33)

Synthesized according to GP2 from 4-chloro-2-fluoro-5-sulfamoylbenzoic acid (48 mg, 0.187 mmol) and 4-chloronaphthalen-2-amine (**65**) (35 mg, 0.187 mmol). Purification of the crude product by preparative HPLC yielded the title compound as a colorless solid (8 mg, 10%). ^1H NMR (500 MHz,

DMSO-d_6) δ 10.20 (s, 1H), 8.52 (s, 1H), 8.16-8.12 (m, 1H), 8.01-7.98 (m, 1H), 7.92 (d, J = 1.8 Hz, 1H), 7.73 (d, J = 2.0 Hz, 1H), 7.65-7.62 (m, 2H), 7.48 (s, 2H), 7.27 (s, 1H). $^{13}C\{^1H\}$ NMR (126 MHz, DMSO-d_6) δ 168.5, 149.6, 136.5, 136.1, 134.6, 133.3, 131.7, 129.7, 128.1, 127.8, 127.6, 126.9, 123.6, 123.5, 119.3, 115.4, 110.7. MALDI-HRMS: *m/z* calculated for $C_{17}H_{12}Cl_2N_2O_4S$[radical]: 409.9889, found: 409.9907.

2-[(4-Bromonaphthalen-2-yl)amino]-4-chloro-5-sulfamoylbenzoic acid (34)

Synthesized according to GP2 from 4-chloro-2-fluoro-5-sulfamoylbenzoic acid (50 mg, 0.194 mmol) and 4-bromonaphthalen-2-amine (50 mg, 0.213 mmol). Purification of the crude product by preparative HPLC yielded the title compound as a colorless solid (12 mg, 14%). ^1H NMR (500 MHz, DMSO-d_6) δ 10.16 (s, 1H), 8.52 (s, 1H), 8.12-8.09 (m, 1H), 8.00-7.97 (m, 2H), 7.91 (d, J = 2.1 Hz, 1H), 7.66-7.60 (m, 2H), 7.49 (s, 2H), 7.26 (s, 1H). $^{13}C\{^1H\}$ NMR (126 MHz, DMSO-d_6) δ 168.5, 149.6, 136.8, 136.1, 134.5, 133.3, 129.7, 128.9, 128.2, 127.7, 127.2, 127.1, 126.2, 122.7, 120.0, 115.4, 110.6. MALDI-HRMS: *m/z* calculated for $C_{17}H_{12}BrClNO_4S$[radical]: 453.9384, found: 453.9381.

2-[(3-Bromophenyl)amino]-4-chloro-5-sulfamoylbenzoic acid (35)

Synthesized according to GP2 from 4-chloro-2-fluoro-5-sulfamoylbenzoic acid (50 mg, 0.193 mmol) and 3-bromoaniline (43 µL, 0.386 mmol). Purification of the crude product by preparative HPLC, followed by flash chromatography (DCM/MeOH) yielded the title compound as a colorless solid (10 mg, 6%). ^1H NMR (500 MHz, DMSO-d_6) δ 13.74 (s, 1H), 10.06 (s, 1H), 8.49 (s, 1H), 7.57 (d, J = 1.3 Hz, 1H), 7.46 (s, 2H), 7.41-7.38 (m, 3H), 7.13 (s, 1H). $^{13}C\{^1H\}$ NMR (126 MHz, DMSO-d_6) δ 168.9, 149.9, 141.1, 136.4, 133.8, 132.0, 130.0, 128.2, 126.3, 122.8, 122.5, 115.6, 111.2. MALDI-HRMS: *m/z* calculated for $C_{13}H_{10}BrClN_2O_4S$[radical]: 403.9228, found: 403.9225.

4-Chloro-2-[(3-iodophenyl)amino]-5-sulfamoylbenzoic acid (36)

Synthesized according to GP2 from 4-chloro-2-fluoro-5-sulfamoylbenzoic acid (50 mg, 0.193 mmol) and 3-iodoaniline (47 µL, 0.386 mmol). Purification of the crude product by preparative HPLC yielded the title compound as a colorless solid (10 mg, 6%). ^1H NMR (500 MHz, DMSO-d_6) δ 13.69 (s, 1H), 9.98 (s, 1H), 8.48 (s, 1H), 7.71 (t, J = 1.8 Hz, 1H), 7.58 (d, J = 7.9 Hz, 1H), 7.46 (s, 2H), 7.40 (dd, J = 8.0, 1.3 Hz, 1H), 7.22 (t, J = 8.0 Hz, 1H), 7.10 (s, 1H). $^{13}C\{^1H\}$ NMR (126 MHz, DMSO-d_6) δ 168.4, 149.5, 140.3, 135.9, 133.7, 133.3, 131.7, 131.5, 129.4, 122.6, 115.0, 110.4, 95.4. MALDI-HRMS: *m/z* calculated for $C_{13}H_{10}IClN_2O_4S$[radical]: 451.9089, found: 451.9084.

2-[(4-Bromophenyl)amino]-4-chloro-5-sulfamoylbenzoic acid (37)

Synthesized according to GP2 from 4-chloro-2-fluoro-5-sulfamoylbenzoic acid (100 mg, 0.386 mmol) and 4-bromoaniline (137 mg, 0.773 mmol). Purification of the crude product by preparative HPLC yielded the title compound as an off-white solid (68 mg, 43%). ^1H NMR (500 MHz, DMSO-d_6) δ 9.95 (s, 1H), 8.49 (s, 1H), 7.61 (d, J = 8.6 Hz, 2H), 7.46 (s, 2H), 7.32 (d, J = 8.9 Hz, 2H), 7.10 (s, 1H). $^{13}C\{^1H\}$ NMR (126 MHz, DMSO-d_6) δ 168.5, 149.7, 138.1, 136.1, 133.3, 132.6, 129.3, 125.5, 117.2, 114.8, 110.1. MALDI-HRMS: *m/z* calculated for $C_{13}H_{10}BrClN_2O_4S$[radical]: 403.9228, found: 403.9227.

4-Chloro-2-[(4-iodophenyl)amino]-5-sulfamoylbenzoic acid (38)

Synthesized according to GP2 from 4-chloro-2-fluoro-5-sulfamoylbenzoic acid (50 mg, 0.193 mmol) and 4-iodoaniline (85 mg, 0.386 mmol). Purification of the crude product by preparative HPLC yielded the title compound as a brown solid (8 mg, 5%). ^1H NMR (500 MHz, DMSO-d_6) δ 13.72 (s, 1H), 10.02 (s, 1H), 8.48 (s, 1H), 7.77-7.75 (m, 2H), 7.45 (s, 2H), 7.19-7.16 (m, 2H), 7.12 (s, 1H). $^{13}C\{^1H\}$ NMR (126 MHz, DMSO-d_6) δ 168.5, 149.5, 138.6, 138.4, 136.0, 133.3, 129.3, 125.4, 114.8, 110.3, 89.2. MALDI-HRMS: *m/z* calculated for $C_{13}H_{10}IClN_2O_4S$[radical]: 451.9089, found: 451.9084.

4-Chloro-2-[(4-ethynylphenyl)amino]-5-sulfamoylbenzoic acid (39)

Synthesized according to GP2 from 4-chloro-2-fluoro-5-sulfamoylbenzoic acid (100 mg, 0.386 mmol) and 4-ethynylaniline (48 µL, 0.425 mmol). Purification of the crude product by preparative HPLC yielded the title compound as a brown solid (20 mg, 15%). ^1H NMR (400 MHz, DMSO-d_6) δ 10.14 (s, 1H), 8.50 (s, 1H), 7.54-7.45 (m, 4H), 7.37-7.32 (m, 2H), 7.22 (s, 1H), 4.16 (s, 1H). $^{13}C\{^1H\}$ NMR (101 MHz, DMSO-d_6) δ 168.4, 149.0, 139.4, 135.9, 133.3, 133.2, 129.6, 122.5, 117.6, 115.2, 110.8, 83.2, 80.6. MALDI-HRMS: *m/z* calculated for $C_{15}H_{11}ClN_2O_4S$[radical]: 350.0123, found: 350.0133.

2-[(3-Bromo-2,4-difluorophenyl)amino]-4-chloro-5-sulfamoylbenzoic acid (40)

Synthesized according to GP2 from 4-chloro-2-fluoro-5-sulfamoylbenzoic acid (100 mg, 0.386 mmol) and 3-bromo-2,4-difluoroaniline (95 µL, 0.773 mmol). Purification of the crude product by flash chromatography (DCM/MeOH) yielded the title compound as a purple solid (98 mg, 57%). ^1H NMR (500 MHz, DMSO-d_6) δ 9.87 (s, 1H), 8.49 (s, 1H), 7.65-7.58 (m, 1H), 7.47 (s, 2H), 7.37-7.32 (m, 1H), 6.84 (d, J = 1.1 Hz, 1H). $^{13}C\{^1H\}$ NMR (126 MHz, DMSO-d_6) δ 168.5, 156.9, 154.1, 150.1, 136.2, 133.2, 129.7, 127.2, 124.0, 114.9, 112.6, 110.2, 98.2. MALDI-HRMS: *m/z* calculated for $C_{13}H_8BrClF_2N_2O_4S$[radical]: 439.9039, found: 439.9034.

2-[(4-Bromo-3,5-difluorophenyl)amino]-4-chloro-5-sulfamoylbenzoic acid (41)

Synthesized according to GP2 from 4-chloro-2-fluoro-5-sulfamoylbenzoic acid (100 mg, 0.386 mmol) and 4-bromo-3,5-difluoroaniline (93 µL, 0.773 mmol). Purification of the crude product by preparative HPLC, followed by flash chromatography (DCM/MeOH) yielded the title compound as a colorless solid (16 mg, 10%). ^1H NMR (500 MHz, DMSO-d_6) δ 12.73-12.60 (m, 1H), 8.57 (d, J = 1.8 Hz, 1H), 7.34-7.09 (m, 6H). $^{13}C\{^1H\}$ NMR (126 MHz, DMSO-d_6) δ 169.1, 159.8, 147.1, 142.3, 133.2, 132.6, 130.1, 120.3, 115.0, 103.7, 88.2. MALDI-HRMS: *m/z* calculated for $C_{13}H_8BrClF_2N_2O_4S$[radical]: 439.9039, found: 439.9035.

2-[(4-Bromo-3-chlorophenyl)amino]-4-chloro-5-sulfamoylbenzoic acid (42)

Synthesized according to GP2 from 4-chloro-2-fluoro-5-sulfamoylbenzoic acid (50 mg, 0.193 mmol) and 4-bromo-3-chloroaniline (47 µL, 0.386 mmol). Purification of the crude product by flash chromatography (DCM/MeOH), followed by preparative HPLC yielded the title compound as a colorless solid (16 mg, 9%). ^1H NMR (500 MHz, DMSO-d_6) δ 13.78 (s, 1H), 9.97 (s, 1H), 8.49 (s, 1H), 7.77 (d, J = 8.6 Hz, 1H), 7.65 (d, J = 2.6 Hz, 1H), 7.48 (s, 2H), 7.30 (dd, J = 8.6, 2.6 Hz, 1H), 7.20 (s, 1H). $^{13}C\{^1H\}$ NMR (126 MHz, DMSO-d_6) δ 168.3, 149.0, 139.8, 136.0, 134.5, 134.0, 133.2, 130.0, 124.7, 123.2, 116.4, 115.6, 110.9. MALDI-HRMS: *m/z* calculated for $C_{13}H_9BrCl_2N_2O_4S$[radical]: 437.8838, found: 437.8835.

2-[(3-Bromo-4-chlorophenyl)amino]-4-chloro-5-sulfamoylbenzoic acid (43)

Synthesized according to GP2 from 4-chloro-2-fluoro-5-sulfamoylbenzoic acid (50 mg, 0.193 mmol) and 3-bromo-4-chloroaniline (47 µL, 0.386 mmol). Purification of the crude product by flash chromatography (DCM/MeOH), followed by preparative HPLC yielded the title compound as a colorless solid (9 mg, 5%). ^1H NMR (500 MHz, DMSO-d_6) δ 13.77 (s, 1H), 10.01 (s, 1H), 8.49 (s, 1H), 7.77 (d, J = 2.5 Hz, 1H), 7.64 (d, J = 8.6 Hz, 1H), 7.48 (s, 2H), 7.42 (dd, J = 8.6, 2.5 Hz, 1H), 7.17 (s, 1H). $^{13}C\{^1H\}$ NMR (126 MHz, DMSO-d_6) δ 168.3, 149.1, 138.2, 136.0, 133.2, 131.1, 130.0, 128.7, 128.1, 123.7, 122.1, 115.4, 110.9. MALDI-HRMS: *m/z* calculated for $C_{13}H_9BrCl_2N_2O_4S$[radical]: 437.8838, found: 437.8834.

4-Chloro-2-[(3,4-dibromophenyl)amino]-5-sulfamoylbenzoic acid (44)

Synthesized according to GP2 from 4-chloro-2-fluoro-5-sulfamoylbenzoic acid (50 mg, 0.193 mmol) and 3,4-dibromoaniline (51 µL, 0.386 mmol). Purification of the crude product by preparative HPLC yielded the title compound as a colorless solid (5 mg, 3%). ^{1}H NMR (500 MHz, DMSO-d$_6$) δ 10.11 (s, 1H), 8.49 (s, 1H), 7.77-7.74 (m, 2H), 7.47 (s, 2H), 7.33 (dd, J = 2.6, 8.6 Hz, 1H), 7.18 (s, 1H). ^{13}C{^{1}H} NMR (126 MHz, DMSO-d$_6$) δ 168.3, 149.0, 139.8, 135.8, 134.4, 133.2, 129.9, 127.8, 124.6, 123.6, 118.7, 115.5, 111.4. MALDI-HRMS: *m/z* calculated for $C_{13}H_9Br_2ClN_2O_4S$[radical]: 483.8312, found: 483.8303.

2-[(3-Bromo-4-iodophenyl)amino]-4-chloro-5-sulfamoylbenzoic acid (45)

Synthesized according to GP2 from 4-chloro-2-fluoro-5-sulfamoylbenzoic acid (100 mg, 0.386 mmol) and 3-bromo-4-iodoaniline (57 µL, 0.425 mmol). Purification of the crude product by preparative HPLC yielded the title compound as a colorless solid (43 mg, 21%). ^{1}H NMR (500 MHz, DMSO-d$_6$) δ 9.96 (s, 1H), 8.48 (s, 1H), 7.92 (d, J = 8.5 Hz, 1H), 7.74-7.70 (m, 1H), 7.48 (s, 2H), 7.20-7.15 (m, 2H). ^{13}C{^{1}H} NMR (126 MHz, DMSO-d$_6$) δ 168.3, 148.9, 140.8, 140.4, 136.0, 133.2, 130.0, 129.7, 126.9, 123.4, 115.6, 110.9, 95.9. MALDI-HRMS: *m/z* calculated for $C_{13}H_9BrClIN_2O_4S$[radical]: 529.8194, found: 529.8188.

4-Chloro-2-[(3-chloro-4-iodophenyl)amino]-5-sulfamoylbenzoic acid (46)

Synthesized according to GP2 from 4-chloro-2-fluoro-5-sulfamoylbenzoic acid (100 mg, 0.386 mmol) and 3-chloro-4-iodoaniline (58 µL, 0.425 mmol). Purification of the crude product by preparative HPLC yielded the title compound as a colorless solid (5 mg, 3%). ^{1}H NMR (500 MHz, DMSO-d$_6$) δ 10.35 (s, 1H), 8.50 (s, 1H), 7.92 (d, J = 8.4 Hz, 1H), 7.56 (s, 1H), 7.46 (s, 2H), 7.20 (s, 1H), 7.11 (d, J = 8.0 Hz, 1H). ^{13}C{^{1}H} NMR (126 MHz, DMSO-d$_6$) δ 168.3, 148.7, 140.8, 140.8, 138.2, 135.5, 133.2, 129.9, 123.2, 122.6, 115.4, 112.2, 92.2. MALDI-HRMS: *m/z* calculated for $C_{13}H_9Cl_2IN_2O_4S$[radical]: 485.8699, found: 485.8694.

4-Chloro-2-[(3-chloro-4-ethynylphenyl)amino]-5-sulfamoylbenzoic acid (47)

Synthesized according to GP2 from 4-chloro-2-fluoro-5-sulfamoylbenzoic acid (100 mg, 0.386 mmol) and 3-chloro-4-ethynylaniline (66 mg, 0.425 mmol). Purification of the crude product by preparative HPLC yielded the title compound as a green solid (57 mg, 38%). ^{1}H NMR (400 MHz, DMSO-d$_6$) δ 10.06 (s, 1H), 8.50 (s, 1H), 7.62-7.49 (m, 4H), 7.36-7.30 (m, 2H), 4.49 (s, 1H). ^{13}C{^{1}H} NMR (101 MHz, DMSO-d$_6$) δ 168.2, 148.3, 140.9, 136.0, 136.0, 134.8, 133.2, 130.5, 122.5, 120.5, 116.8, 116.2, 111.6, 85.5, 80.0. MALDI-HRMS: *m/z* calculated for $C_{15}H_{10}Cl_2N_2O_4S$[radical]: 383.9733, found: 383.9742.

4-Chloro-2-[(3-chloro-4-cyanophenyl)amino]-5-sulfamoylbenzoic acid (48)

Synthesized according to GP2 from 4-chloro-2-fluoro-5-sulfamoylbenzoic acid (100 mg, 0.386 mmol) and 4-amino-2-chlorobenzonitrile (50 µL, 0.425 mmol). Purification of the crude product by flash chromatography (DCM/MeOH), followed by preparative HPLC yielded the title compound as a colorless solid (33 mg, 22%). ^{1}H NMR (400 MHz, DMSO-d$_6$) δ 10.11 (s, 1H), 8.51 (s, 1H), 7.88 (d, J = 8.6 Hz, 1H), 7.65 (d, J = 2.0 Hz, 1H), 7.59-7.52 (m, 3H), 7.45 (dd, J = 8.6, 2.0 Hz, 1H). ^{13}C{^{1}H} NMR (101 MHz, DMSO-d$_6$) δ 167.8, 146.5, 145.6, 136.7, 135.9, 135.6, 132.9, 132.3, 120.6, 118.6, 118.4, 116.4, 114.0, 105.0. MALDI-HRMS: *m/z* calculated for $C_{14}H_9Cl_2N_3O_4S$[radical]: 384.9685, found: 384.9694.

4-Chloro-2-((4-iodo-3-methylphenyl)amino)-5-sulfamoylbenzoic acid (49)

Synthesized according to GP2 from 4-chloro-2-fluoro-5-sulfamoylbenzoic acid (400 mg, 1.55 mmol) and 4-iodo-3-methylaniline (238 µL, 1.70 mmol). Purification of the crude product by flash chromatography (DCM/MeOH), followed by preparative HPLC yielded the title compound as a

colorless solid (32 mg, 4%). HPLC-purity (254 nm): >99%. ^{1}H NMR (500 MHz, DMSO-d_6) δ 10.09 (s, 1H), 8.48 (s, 1H), 7.83 (d, J = 8.4 Hz, 1H), 7.44 (s, 2H), 7.32 (d, J = 2.4 Hz, 1H), 7.12 (s, 1H), 6.97 (dd, J = 8.3, 2.6 Hz, 1H), 2.38-2.37 (s, 3H). $^{13}C\{^{1}H\}$ NMR (126 MHz, DMSO-d_6) δ 168.5, 149.6, 142.5, 139.6, 139.0, 135.9, 133.3, 129.1, 124.7, 122.5, 114.8, 110.5, 95.8, 27.4. MALDI-HRMS: *m/z* calculated for $C_{14}H_{12}ClIN_2O_4S$[radical]: 465.9246, found: 465.9241.

4-Chloro-2-((4-cyano-3-methylphenyl)amino)-5-sulfamoylbenzoic acid (50)

Synthesized according to GP2 from 4-chloro-2-fluoro-5-sulfamoylbenzoic acid (100 mg, 0.386 mmol) and 4-amino-2-methylbenzonitrile (56 mg, 0.425 mmol). Purification of the crude product by flash chromatography (DCM/MeOH), followed by preparative HPLC yielded the title compound as a colorless solid (35 mg, 25%). ^{1}H NMR (400 MHz, DMSO-d_6) δ 10.16 (s, 1H), 8.51 (s, 1H), 7.74 (d, J = 8.4 Hz, 1H), 7.53 (s, 2H), 7.41-7.38 (m, 2H), 7.32 (d, J = 8.3 Hz, 1H), 2.46 (s, 3H). $^{13}C\{^{1}H\}$ NMR (101 MHz, DMSO-d_6) δ 168.3, 147.7, 143.5, 143.5, 136.0, 134.1, 133.1, 131.0, 122.0, 118.5, 118.1, 116.8, 112.2, 106.1, 20.0. MALDI-HRMS: *m/z* calculated for $C_{15}H_{12}ClN_3O_4S$[radical]: 365.0232, found: 365.0232.

2-((3-Chlorophenyl)amino)benzonitrile (51)

2--Bromobenzonitrile (150 mg, 0.824 mmol), 3-chloroaniline (105 µL, 0.989 mmol), NaOMe (111 mg, 1.15 mmol), Pd2(dba)3 (38 mg, 0.0412 mmol) and rac-BINAP (16 mg, 0.0247) were suspended in toluene (1.5 mL) and heated in an oil bath to 90 °C overnight. After that time, the reaction mixture was filtrated over celite. Reaction mixture was diluted with EtOAc and the organic phase was washed 2 x with H2O, 1 x with an aq saturated NaCl solution, dried over MgSO4, and concentrated under reduced pressure. Purification of the crude product by column chromatography (n-hexane/EtOAc) yielded the title compound as a brown solid (51 mg, 27%). 1H NMR (250 MHz, acetone-d6) δ 10.14 (s, 1H), 7.80-7.77 (m, 1H), 7.39-7.37 (m, 2H), 7.37-7.26 (m, 1H), 7.22-7.21 (m, 1H), 7.15-7.11 (m, 1H), 6.98-6.95 (m, 1H), 6.90-6.83 (m, 1H).

DNA and protein sequence of msEH-P-p7XNH3

DNA sequence of msEH-P-p7XNH3 (10xHis-Tag_3C-protease-cleavage-site_msEH-P):

```
ATGCACCATCATCATCACCATCATCATCATCATTTAGAAGTTTTGTTTCAAGGTCCAAGTGCGCTGCGTGTAGCC
GCGTTCGACCTTGACGGAGTGCTGGCCCTCCCCTCTATCGCCGGGGCTTTCCGCCGCAGCGAAGAGGCCCTGGCA
CTGCCTAGAGACTTCCTGCTTGGTGCGTACCAGACGGAATTCCCAGAGGGACCCACTGAGCAACTCATGAAAGGG
AAGATCACATTTTCGCAGTGGGTACCACTCATGGATGAAAGCTACAGGAAGTCCTCCAAAGCCTGTGGAGCCAAT
CTACCTGAGAATTTCTCCATAAGTCAAATATTCAGCCAAGCTATGGCAGCAAGAAGCATCAACCGCCCCATGCTT
CAGGCAGCCATTGCTCTCAAAAAGAAAGGATTCACAACATGCATTGTCACCAACAACTGGCTGGACGACGGAGAC
AAGAGAGACAGCCTGGCCCAGATGATGTGTGAGCTGAGCCAACACTTTGACTTCCTGATAGAGTCCTGTCAGGTT
GGGATGATCAAGCCTGAGCCTCAGATCTACAATTTTTTACTGGATACCCTGAAGGCAAAACCCAATGAGGTTGTT
TTCCTAGATGACTTTGGAAGTAATCTGAAGCCAGCCCGTGACATGGGGATGGTTACCATCCTGGTCCACAACACA
GCCTCCGCTCTGAGAGAACTGGAGAAGGTCACAGGGACACAGTTTCCTGAGGCCGCA
```

Protein sequence of msEH-P-p7XNH3 (10xHis-Tag_3C-protease-cleavage-site_msEH-P):

```
MHHHHHHHHHHLEVLFQGPSALRVAAFDLDGVLALPSIAGAFRRSEEALALPRDFLLGAYQTEFPEGPTEQLMKG
KITFSQWVPLMDESYRKSSKACGANLPENFSISQIFSQAMAARSINRPMLQAAIALKKKGFTTCIVTNNWLDDGD
KRDSLAQMMCELSQHFDFLIESCQVGMIKPEPQIYNFLLDTLKAKPNEVVFLDDFGSNLKPARDMGMVTILVHNT
ASALRELEKVTGTQFPEAA
```

Supplementary Tables

Supplementary Table 1. Mass spectrometry parameters for FZ581, LPAs and epoxyfatty acids.

Analyte	Mass transition		MS parameters				Adduct	Retention time (min)
	m/z (MS1)	m/z (MS3)	Dwell Time (msec)	Q1 Pre Bias (V)	CE	Q3 Pre Bias (V)		
FZ581	464.90	420.90	4	17	20	22	$[M\text{-}H]^-$	2.358
LPA (16:0)	409.10	152.90	8	15	23	17	$[M\text{-}H]^-$	6.082
LPA (18:0)	437.30	152.90	8	17	24	17	$[M\text{-}H]^-$	6.891
LPA (18:1)	435.20	152.90	8	30	25	26	$[M\text{-}H]^-$	6.259
LPA (17:1)	421.20	152.90	8	16	23	11	$[M\text{-}H]^-$	5.789
14,15-EET	319.00	219.25	8	17	12	16	$[M\text{-}H]^-$	5.739
14,15-EET-d11	330.10	219.25	5	16	12	16	[M-H]-	5.682
14,15-EpETE	317.20	248.20	17	15	11	18	$[M\text{-}H]^-$	5.158
16,17-EpDPA	343.20	274.10	17	16	12	11	$[M\text{-}H]^-$	5.973

Analysis: Non-compartmental pharmacokinetic analysis (NCA) of FZ581 was performed using PKAnalix from MonolixSuite version 2023R1 (Lixoft, Antony, France). Available plasma levels of FZ581 following extravascular administrations were used (40 and 28 samples for rats and mice respectively). Terminal elimination rate constant (λz) for each rodent was computed by using the last 2 to 4 sampling points according to adjusted coefficient of determination (adj. R^2). The area under the concentration-time curves (AUC) were calculated by linear trapezoidal method.

Statistical analysis of the evolution of LPAs after administration of FZ581 was performed by repeated-measures ANOVA and the differences between FZ581 and vehicle for the epoxyfatty acids were analyzed by ANOVA followed by Bonferroni post hoc tests using GraphPad Prism software (version 8.0.2).

Supplementary Table 2. Summary of non-compartmental analysis (NCA) parameters.

TYPE	Metrics	N	mean	SD	CV(%)	min	median	max
Mice 0.1 mg/kg IV	$AUC_{0\text{-}inf}$/Dose	2	66.2	5.15	7.78	62.56	66.2	69.84
	$AUC_{0\text{-}inf}$	2	201.74	11.02	5.46	193.95	201.74	209.53
	$AUC_{0\text{-}tlast}$	2	161.12	7.61	4.72	155.74	161.12	166.5
	$AUC_{0\text{-}tlast}$/Dose	2	52.87	3.72	7.03	50.24	52.87	55.5
	C_{max}	2	98.45	31.61	32.11	76.1	98.45	120.8
	C_{max}/Dose	2	32.17	9.62	29.9	25.37	32.17	38.97
	k_{el}	2	0.58	0.25	42.68	0.4	0.58	0.75
Mice 1 mg/kg p.o.	$AUC_{0\text{-}inf}$/Dose	7	11.17	3.58	32	6.18	12.8	14.99
	$AUC_{0\text{-}inf}$	7	291.05	103.53	35.57	166.82	298.17	422.51
	$AUC_{0\text{-}tlast}$	7	185.95	68.01	36.58	122.91	149.68	297.06
	$AUC_{0\text{-}tlast}$/Dose	7	7.16	2.51	35.09	4.24	5.99	10.61
	C_{max}	7	75.9	24.51	32.3	31.2	86.5	100.9
	C_{max}/Dose	7	2.94	1.03	34.92	1.3	3.11	4.07
	k_{el}	7	0.23	0.12	50.14	0.11	0.2	0.46

Mice 10 mg/kg p.o.	$AUC_{0\text{-}inf}$/Dose	3	5.43	1.67	30.67	4.36	4.58	7.35
	$AUC_{0\text{-}inf}$	3	1569.34	551.05	35.11	1221.44	1281.9	2204.68
	$AUC_{0\text{-}tlast}$	3	794.8	133.69	16.82	662	793.05	929.36
	$AUC_{0\text{-}tlast}$/Dose	3	2.76	0.37	13.43	2.36	2.83	3.1
	C_{max}	3	389.3	56.57	14.53	336.5	382.4	449
	C_{max}/Dose	3	1.35	0.15	10.91	1.2	1.37	1.5
	k_{el}	3	0.19	0.039	20.72	0.16	0.17	0.23
Rat 1 mg/kg p.o.	$AUC_{0\text{-}inf}$/Dose	3	15.98	6.09	38.12	9.05	18.43	20.47
	$AUC_{0\text{-}inf}$	3	7687.84	2816.26	36.63	4477.46	8844.21	9741.84
	$AUC_{0\text{-}tlast}$	6	4969.25	1874.39	37.72	3134.25	4655.25	7760.25
	$AUC_{0\text{-}tlast}$/Dose	6	10.26	3.86	37.65	6.58	9.65	16.17
	C_{max}	6	48.68	48.74	100.11	12.8	24.55	131.4
	C_{max}/Dose	6	0.1	0.1	100.5	0.027	0.051	0.27
	k_{el}	3	0.0088	0.0067	76.77	0.001	0.013	0.013

Supplementary Figures

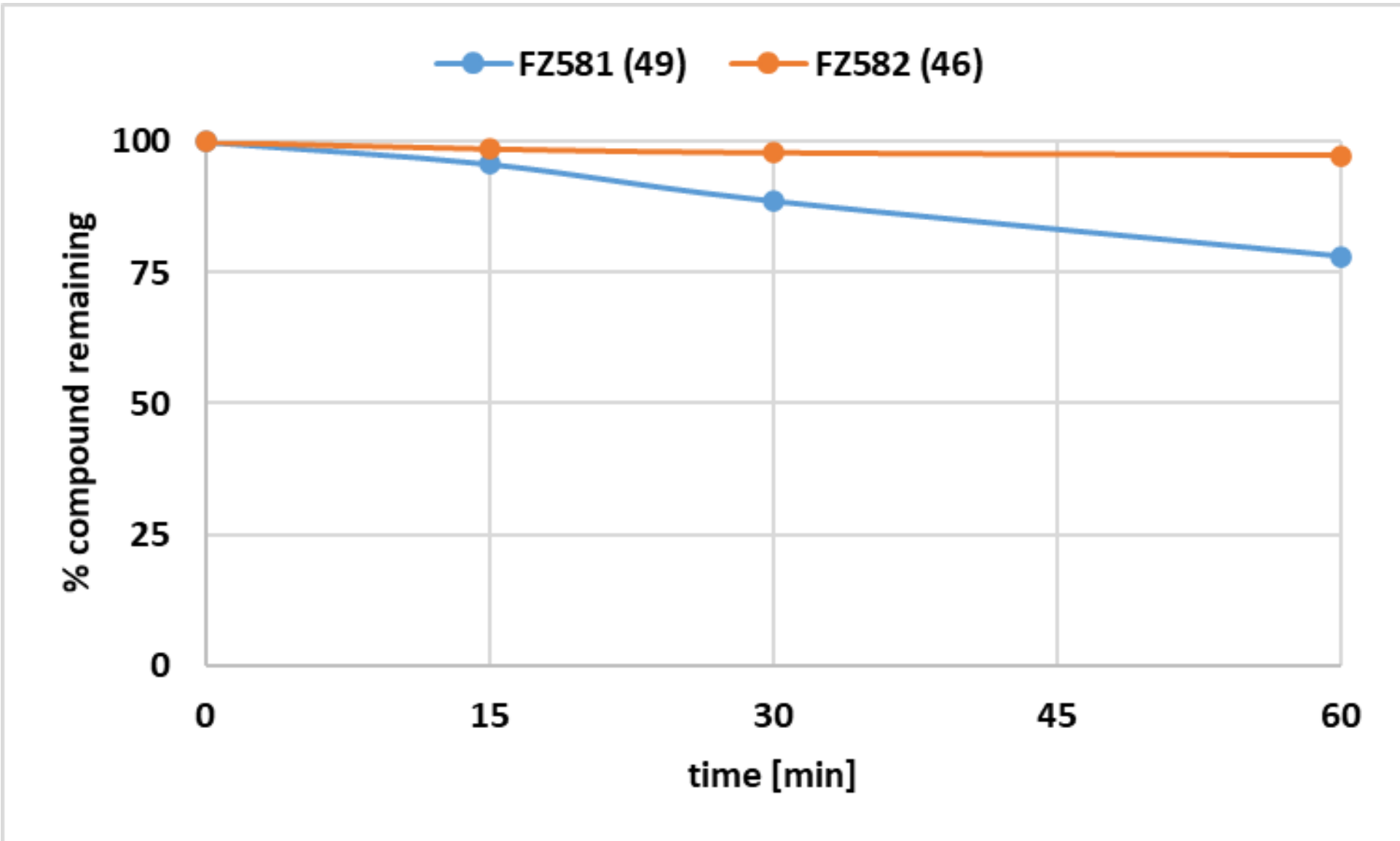

Supplementary Figure 1. Stability of compound **49** and **46** in rat liver microsomes (n = 3).

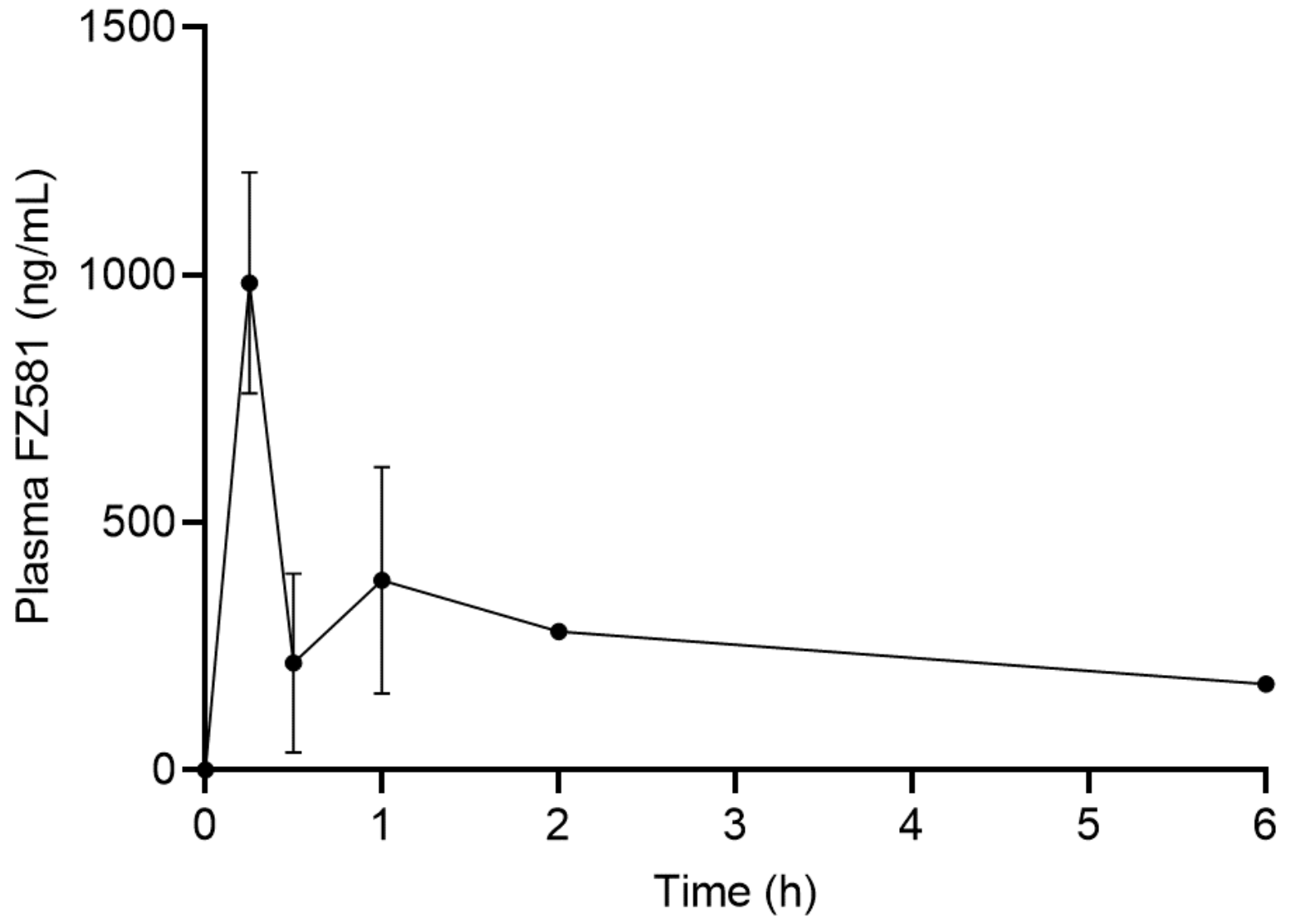

Supplementary Figure 2. time course of of plasma levels of FZ581 in C57BL/6 mice (n = 4) during 6 hours after intravenous administration at 1 mg/kg.

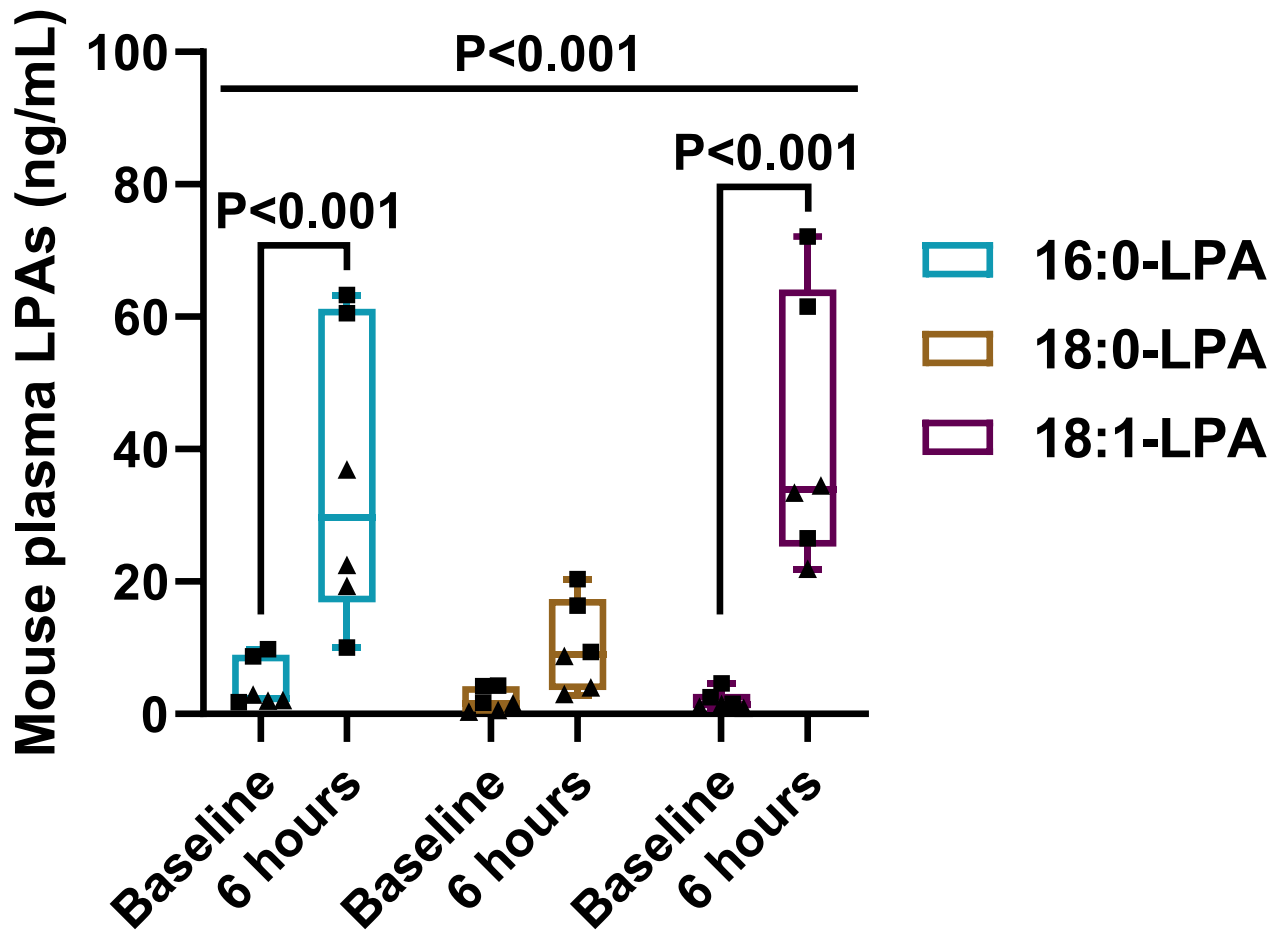

Supplementary Figure 3. Plasma levels of 1-palmitoyl LPA (sn1-16:0 LPA), 1-steraoyl LPA (sn1-18:0 LPA) and 1-oleyl LPA (sn1-18:1 LPA) at baseline and 6 hours after administration of FZ581 (60) at 1 mg/kg (square) and 10 mg/kg (triangle) in rats.

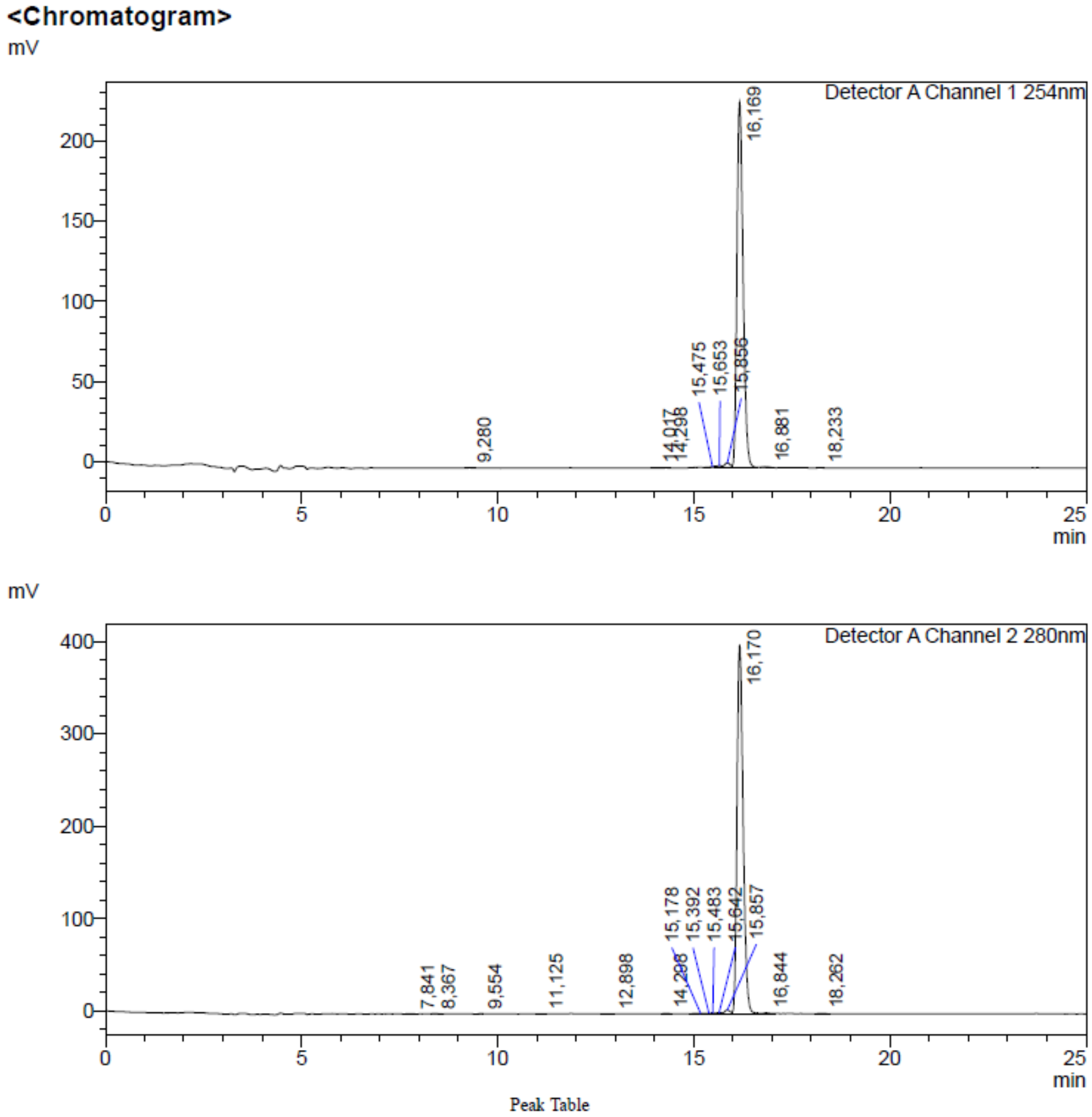

Supplementary Figure 4. HPLC Chromatogram of FZ581.

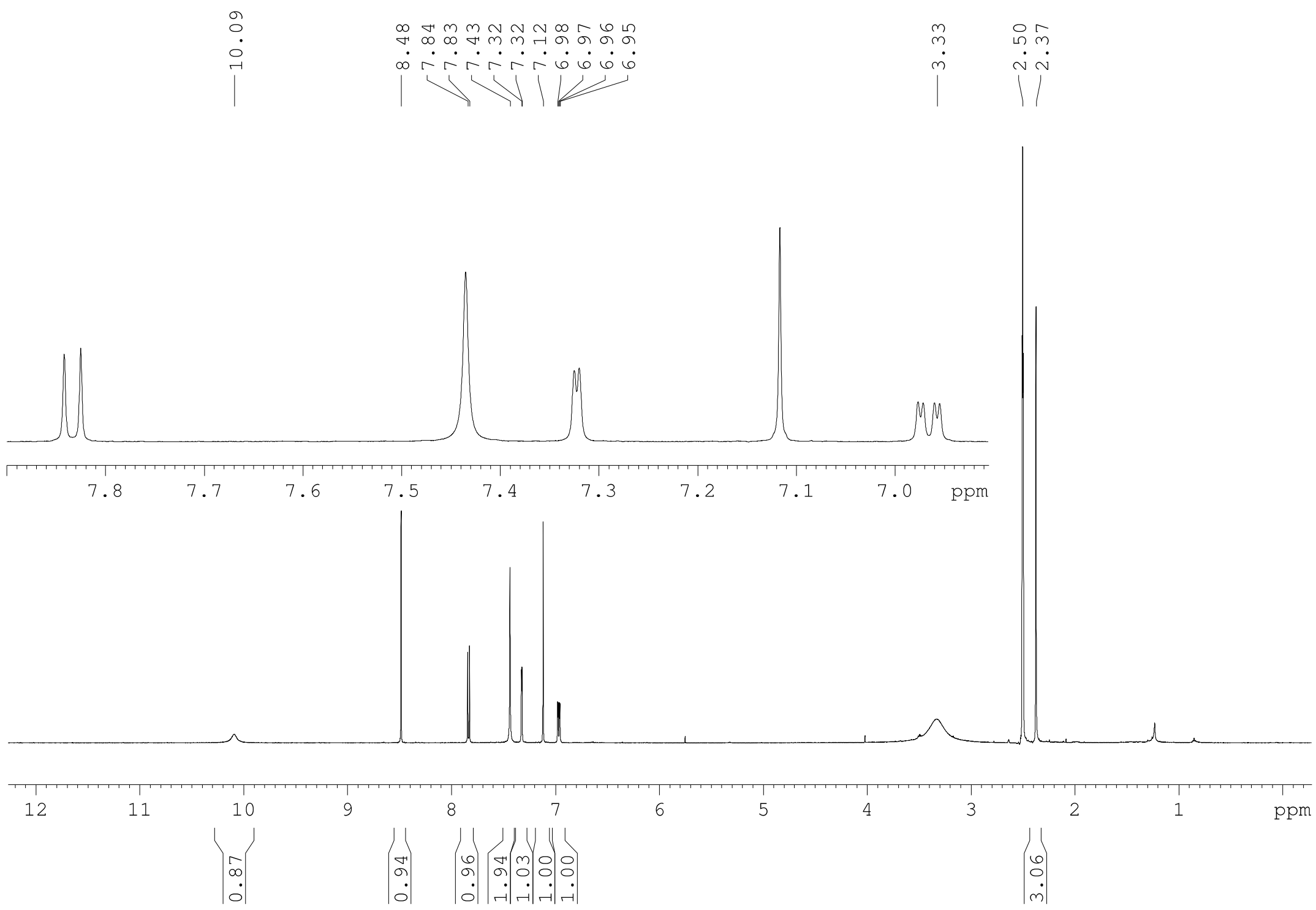

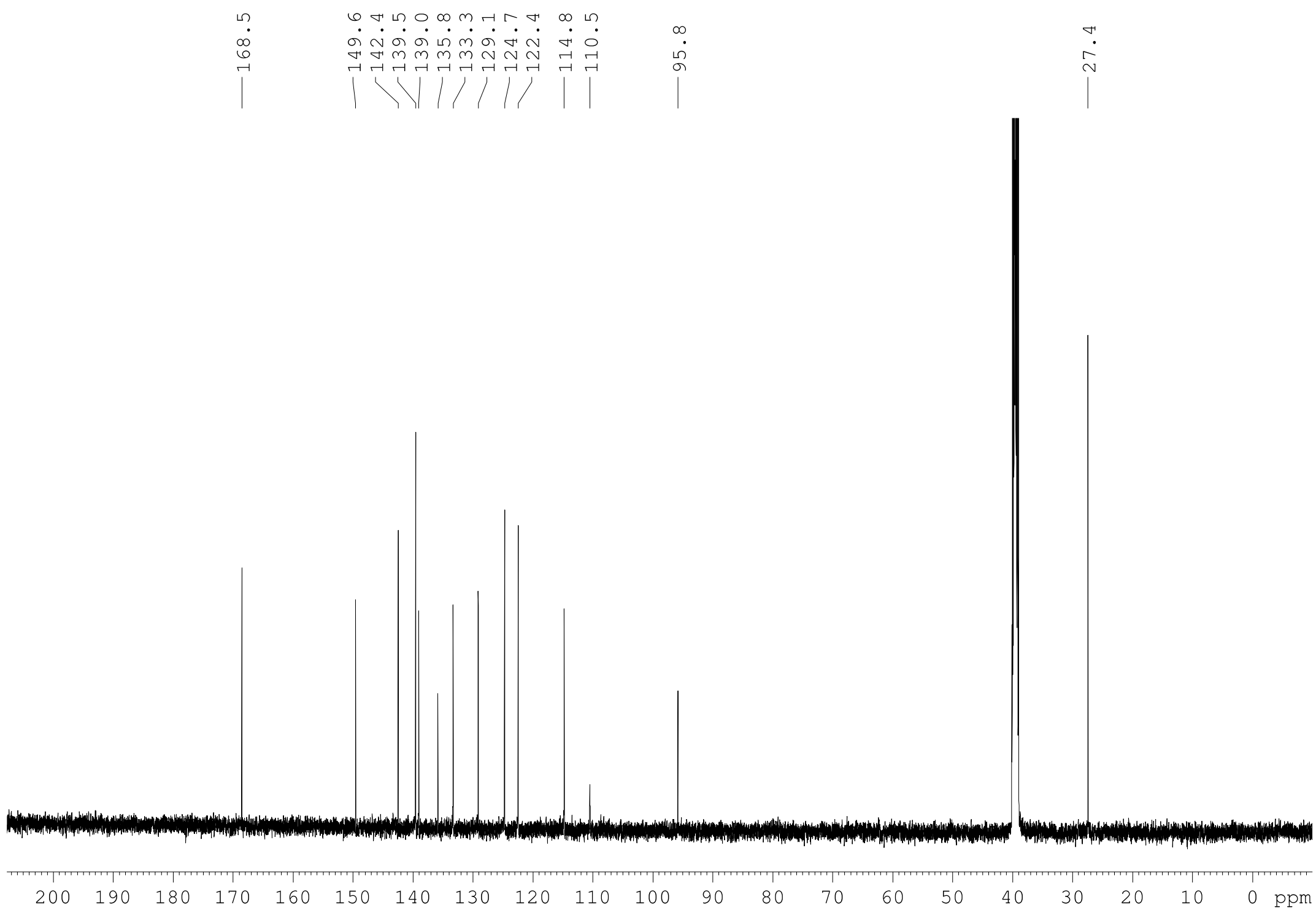

Supplementary Figure 5. NMR spectra of FZ581.
